# Monkeypox outbreaks in the context of the COVID-19 pandemic: Network and clustering analyses of global risks and modified SEIR prediction of epidemic trends

**DOI:** 10.3389/fpubh.2023.1052946

**Published:** 2023-01-24

**Authors:** Jing Gao, Cui Zhou, Hanwei Liang, Rao Jiao, Åsa M. Wheelock, Kedi Jiao, Jian Ma, Chutian Zhang, Yongman Guo, Sitong Luo, Wannian Liang, Lei Xu

**Affiliations:** ^1^Vanke School of Public Health, Tsinghua University, Beijing, China; ^2^Institute for Healthy China, Tsinghua University, Beijing, China; ^3^Respiratory Medicine Unit, Department of Medicine and Centre for Molecular Medicine, Karolinska Institute, Stockholm, Sweden; ^4^Heart and Lung Centre, Department of Pulmonary Medicine, University of Helsinki and Helsinki University Hospital, Helsinki, Finland; ^5^Department of Mathematical Science, Tsinghua University, Beijing, China

**Keywords:** monkeypox, global health, risk assessment, prediction, COVID-19, SEIR

## Abstract

**Background:**

Ninety-eight percent of documented cases of the zoonotic disease human monkeypox (MPX) were reported after 2001, with especially dramatic global spread in 2022. This longitudinal study aimed to assess spatiotemporal risk factors of MPX infection and predict global epidemiological trends.

**Method:**

Twenty-one potential risk factors were evaluated by correlation-based network analysis and multivariate regression. Country-level risk was assessed using a modified Susceptible-Exposed-Infectious-Removed (SEIR) model and a risk-factor-driven k-means clustering analysis.

**Results:**

Between historical cases and the 2022 outbreak, MPX infection risk factors changed from relatively simple [human immunodeficiency virus (HIV) infection and population density] to multiple [human mobility, population of men who have sex with men, coronavirus disease 2019 (COVID-19) infection, and socioeconomic factors], with human mobility in the context of COVID-19 being especially key. The 141 included countries classified into three risk clusters: 24 high-risk countries mainly in West Europe and Northern America, 70 medium-risk countries mainly in Latin America and Asia, and 47 low-risk countries mainly in Africa and South Asia. The modified SEIR model predicted declining transmission rates, with basic reproduction numbers ranging 1.61–7.84 in the early stage and 0.70–4.13 in the current stage. The estimated cumulative cases in Northern and Latin America may overtake the number in Europe in autumn 2022.

**Conclusions:**

In the current outbreak, risk factors for MPX infection have changed and expanded. Forecasts of epidemiological trends from our modified SEIR models suggest that Northern America and Latin America are at greater risk of MPX infection in the future.

## Introduction

Monkeypox (MPX) is a zoonotic infection caused by the MPX virus (MPVX) (1, 2). Direct contact with infected human or animal secretions or contaminated objects is the main transmission route for monkeypox virus (MPXV) (3, 4). In the several decades over which cases have been documented, human MPX infections have primarily been reported in 16 countries in West and Central Africa, and patients in other continents have usually had a history of travel to the endemic area (5–7). However, a dramatic increase and global spread of human MPX infections occurred in 2022, triggering the first multi-country outbreak of MPX in non-African countries and becoming a new global health emergency (8, 9). The World Health Organization (WHO) International Health Regulations Multinational Outbreak Committee on Monkeypox Outbreaks expressed concern about the MPX outbreak on 23 June 2022 (10). As of 16 August 2022, ~37,000 MPX cases had been reported on six continents, with the majority of involved countries reporting MPX infections for the first time (11). In this current outbreak, MPX is spreading dramatically outside of its historically suitable geographic settings and affecting many more people outside Africa (5, 12). As such, prior knowledge of MPX is no longer sufficient to explain the current epidemic. Moreover, with the public health and economic crisis caused by coronavirus disease 2019 (COVID-19) still raging, the current MPX outbreak may exacerbate the burden on health care systems and lead to “combination virus” hazards (5, 13). Therefore, there is an urgent need to assess emerging risks of MPX infection in the context of the COVID-19 pandemic so as to guide countries in developing response strategies.

Notably, with the current MPX outbreak, transmission patterns and susceptible populations may be changing relative to historical trends. Human mobility is emerging as an important factor in cross-regional MPX transmission (14); moreover, distinct from previous outbreaks, this outbreak has seen sustained human-to-human transmission (7, 15). In the post-COVID-19 era, as national restrictions end, most countries around the world have returned to their pre-pandemic lifestyles (16), and international travel and mass gatherings are gradually resuming. Therefore, it is necessary to explore changes in human mobility and MPX transmission patterns in a timely manner. In addition, where previous studies indicated children under 15 years of age as historically being at highest risk of MPX infection, with no significant difference in gender among patients (17), MPX cases reported in recent studies mainly involve but are not limited to males aged 20–50, with many patients being men who have sex with men (MSM) (6, 15, 18). Although current evidence is uncertain as to whether sexual behavior may have made MPX more likely to spread, there is need for an assessment of the potential risk of MPX infections among MSM to protect this potential high-risk population (19–21). In a third consideration, MPXV and the smallpox virus are both orthopoxviruses and have cross-immunity with one another (22); with the vaccination against smallpox having been phased out globally around 1980, people under the age of 40 worldwide are likely to be at high risk of MPX infection (23, 24). Finally, MPX has repeatedly shown associations with demographic characteristics (23), forest cover (1, 25), temperature (1), precipitation (25–27), socioeconomic factors (28, 29), biodiversity (30), and health-associated factors, which are also potential risk factors for most infectious diseases.

Here, we aimed to identify risk factors for MPX infection and changes in predominant factors over time by examining multidimensional characteristics that potentially affect MPX. We further evaluated the risk of MPX in different spatial and temporal contexts using correlation-based networks and multivariate regression, and also assessed country-level risk based on a modified susceptibility-exposure-infection removal (SEIR) model and a clustering algorithm.

## Materials and methods

### Overview

In this study, we first clarified changes in risk factors for MPX infection from the past to the present, and then identified key risk factors for the current outbreak in 2022. We further constructed a SEIR model based on those key risk factors to predict the scale of the epidemic in the future and to calculate the basic reproduction number (*R*_0_) of MPX across continents. Also, we assessed country-level risk in the 2022 epidemic through key-risk-factor-driven *k*-means clustering analysis and characterized the countries in each identified cluster. This study complies with the Guidelines for Accurate and Transparent Health Estimates Reporting (GATHER) recommendations (Supplementary Table 1). Analyses were performed in the R 4.1.1 and Python 3.8 environments. All maps were exported from ArcGIS 10.7.

### Data sources

#### Public data set of MPX

The public dataset used in this study comprises the country-level counts of individuals reported with MPX from 1970 to 2022, and most of cases reported since 2001. The cumulative number of MPX cases and also daily new cases were obtained from the Global Infectious Disease and Epidemiology Network (GIDEON) (31), the Global Health team^1^, and the literature (23). Countries with MPX cases were divided into endemic and external-imported countries with reference to a WHO report (6) (Supplementary Table 2). MPX prevalence was mainly summarized over two time periods: historical infections (2001–2021) and the current epidemic (31 January−16 August 2022). Imported and endemic country distributions was mapped.

#### Multi-dimensional explanatory variables

We included 21 features of seven dimensions that are known or thought to affect MPX. These consisted of socio-economic factors: population density (32), aged under 40 years (33), education level (34), gross domestic product (GDP) (35), GDP growth (36), legal same-sex marriage (37), and MSM population size (38); MPXV-associated biodiversity (39); environmental risk factors: forest area (40), precipitation, and temperature (41); factors relating to national health burden: human immunodeficiency virus (HIV) infection (42) and COVID-19 infection (43); behavioral risk factors: overweight (44) and low physical activity (45); level of national health services: healthcare access and quality index (HAQ) (46), international health regulations index (IHR) (47), and health expenditure (48); and factors reflecting mobility: inbound travelers (49), air passengers (50), and international flight arrivals^2^ (Table 1; Supplementary Figure 2; Supplementary Table 3). When analyzing the 2022 outbreak, data for all explanatory variables were from the most recent year, data on COVID-19 cases spanned 1 January to 16 August 2022, and data on international flight arrivals were from April and May 2022. When analyzing data from 2001 to 2021, all explanatory variables from each country were averaged across the study period. MPXV-associated biodiversity was measured as the abundance of mammalian species that can be infected by MPVX (Supplementary Figure 2; Supplementary Table 4; Supplementary File 1). Details concerning each variable in the analyses and models are given in Table 1.

**Table 1:** List of variables used in this study^*^.

**Variable**	**Value [median (IQR)]**		
	**2001–2021**	**2022**	
	**All (*****n*** = **11)**	**Endemic (*****n*** = **11)**	**Imported (*****n*** = **80)**
**Socioeconomic factors**			
Population density (people per sqkm of land area)	464.0 (27.2–235.0)	56.2 (27.8–109.0)	93.0 (31.8–220.0)
Aged under 40 (%)	80.0 (57.3–81.9)	81.7 (80.1–82.5)	51.2 (46.0–63.7)
Education (years)	5.9 (4.2–11.9)	6.2 (4.5–6.6)	11.3 (9.6–12.4)
GDP per capita (thousands US$)	2.1 (0.5–39.2)	1.5 (0.6–2.2)	17.7 (7.7–43.3)
GDP growth (%)	−0.2 (−0.8 to 1.6)	0.4 (−1.9 to 1.3)	−5.2 (−7.8 to −3.0)
MSM population size (thousands)	85.8 (3.2–195.0)	30.9 (3.9–70.0)	88.0 (34.0–275.0)
Legal same sex marriage (*n*, %)	2 (18.2)	0	31 (38.8)
**Environment**			
Forest area (%)	33.8 (18.1–47.2)	35.8 (31.5–60.0)	33.6 (18.6–47.2)
Temperature (°C)	26.1 (24.8–26.9)	26.4 (25.4–27.1)	14.8 (10.1–24.4)
Precipitation (mm per year)	1,190.0 (850.0–1,890.0)	1,530.0 (1,050.0–1,870.0)	900.0 (631.0–1,390.0)
**Biodiversity**			
MPXV (species number)	3.0 (0.5–6.0)	7.0 (5.0–7.0)	1.0 (0–1.0)
**Disease burden**			
HIV (*n*, thousand)	101.0 (53.3–329.0)	104.0 (72.9–409.0)	12.6 (1.9–73.6)
COVID-19 (*n*, thousand)	46.2 (11.1–536.0)	5.8 (2.3–11.1)	839.0 (277.0–2,820.0)
**Health service**			
HAQ	48.5 (38.4–87.1)	32.2 (31.0–39.3)	81.9 (68.5–919.0)
IHR	55.7 (52.5–88.2)	49.0 (38.5–51.0)	79.0 (67.0–88.3)
Expenditure (per capita in US$)	69.2 (46.6–1,940.0)	52.6 (41.7–73.3)	1,360.0 (545.0–3,640.0)
**Health behavior**			
Overweight (*n*, %)	26.7 (23.9–52.2)	30.9 (28.3–31.8)	58.4 (55.8–62.3)
Low physical activity (DALY, years)	203.0 (165.0–242.0)	208.0 (179.0–239.0)	165.0 (131.0–238.0)
**Mobility**			
Inbound travelers (*n*, millions)	21.9 (14.7–58.1)	0.7^**^	2.5 (0.8–7.3)
Air passengers (*n*, millions)	3.3 (0.4–30.6)	0.3 (0.2–0.3)	6.2 (0.8–25.0)
International flight arrivals (*n*, millions)	NA	0.1 (0.03–0.1)	1.0 (0.4–2.5)

Characteristics are presented as medians with interquartile ranges (IQR) for continuous variables and counts with percentages for categorical variables unless otherwise stated. MPX, monkeypox infections; GDP, gross domestic product; MSM, men who have sex with men; MPXV, monkeypox virus; HIV, human immunodeficiency virus; COVID-19, coronavirus disease 2019; HAQ, healthcare access and quality Index; IHR, international health regulations; WHO, world health organization; GBD, global burden of disease Study; DALY, disability adjusted life year; NA, not available.

* Data resources and a detailed explanation of variables are presented in Supplementary Table 3.

** Due to missing data for ten countries, only one value is available for this variable.

### Gray model

In predicting the future of MPX dynamics, the original gray model with a single-variable and first-order equation [GM (1, 1)] was used (51, 52). When building the model, the number of global annual recorded MPX cases from 2001 to 2021 was used to predict the number of cases from 2022 to 2024. Formulas are detailed in Supplementary Files 2, 3.

### Correlation-based network analysis and multivariate regression model

To explore risk factors for MPX, we conducted correlation-based network analyses and constructed multivariate regression models. Risk factors were compared among three networks: (1) all countries in the current outbreak, (2) imported countries in the current outbreak, and (3) all countries in history (2001–2021). First, Spearman's rank correlation was used to assess the association between MPX infection and multidimensional explanatory variables so as to identify comprehensive risk factors (association coefficient *r* < 0.3, *p* < 0.05). Next, network analysis was performed on pairs of factors, with edge widths representing the significance of connections [–log10(*p*-value)]. Factors were then classified into groups using the leading eigenvector method in the Python package igraph (53). Highly associated variables were classified into one group, and those highly associated with MPX were classified into the MPX risk group and identified as main risk factors.

### Multivariate regression model

Multivariable regression was applied to the identified main factors to distinguish key risk factors. Negative binomial regression was used to identify associations, covariates with *p*-values >0.05 were removed from the final model, and the Akaike information criterion (AIC) was used to select the model that best fit the data.

### Clustering analysis models

The *k*-means algorithm was used to divide countries into clusters based on the above-identified comprehensive risk factors of MPX infections, a demarcation with potential utility in risk stratification practice (54). This algorithm partitions samples into groups of equal variance by minimizing the inertia criterion. We examined *k*-values of 1–9, and identified the optimal number of clusters based on the gap statistic (Supplementary Figure 3). Country clustering was mapped.

### Modified SEIR model

The SEIR model was used to predict the size of MPX outbreaks on six continents in 2022 (55). Specifically, we built a modified SEIR model (Flight-SEIR model) (56) that considers international flight arrival dynamics and susceptible population size and fits a time-dependent parameter (transmission rate) according to changes of policy (57). *R*_0_ is calculated by dividing the transmission rate by the recovery rate (58). Our modified SEIR model is as follows:

If earlier than 23 June 2022:


$$\begin{array}{lll} \frac{dS_{i}}{dt} & = & -\frac{\beta_{i1}S_{i}I_{i}}{n_{i}}+\underset{i}{\sum }S_{ji}-\underset{i}{\sum }S_{ij}\\ \frac{dE_{i}}{dt} & = & \frac{\beta_{i1}S_{i}I_{i}}{n_{i}}-\sigma E_{i}+\underset{i}{\sum }E_{ji}-\underset{i}{\sum }E_{ij}\\ \frac{dI_{i}}{dt} & = & \sigma E_{i}-\gamma I_{i}\\ \frac{dR_{i}}{dt} & = & \gamma I_{i} \end{array}$$

Else:


$$\begin{array}{lll} \frac{dS_{i}}{dt} & = & -\frac{\beta_{i2}S_{i}I_{i}}{n_{i}}+\underset{i}{\sum }S_{ji}-\underset{i}{\sum }S_{ij}\\ \frac{dE_{i}}{dt} & = & \frac{\beta_{i2}S_{i}I_{i}}{n_{i}}-\sigma E_{i}+\underset{i}{\sum }E_{ji}-\underset{i}{\sum }E_{ij}\\ \frac{dI_{i}}{dt} & = & \sigma E_{i}-\gamma I_{i}\\ \frac{dR_{i}}{dt} & = & \gamma I_{i}\\ S_{ij} & = & Passengers_{ij}\times \frac{S_{i}}{N_{i}}\\ E_{ij} & = & Passengers_{ij}\times \frac{E_{i}}{N_{i}} \end{array}$$

The population is divided into different groups based on infection status: susceptible (S), exposed (infected but not yet infectious) (E), infectious (I), and recovered (R). The variables β, σ, and γ represent transmission, incubation, and recovery rate, respectively. The parameters used in this model are summarized in Supplementary Table 6.

The above model adds several improvements over the base SEIR. Firstly, it incorporates data on national flight arrivals. We simulated the number of international flight arrivals per continent from 31 January 2022 to 31 December 2022 based on the number reported from 1 April to 31 May. Specifically, each flight's departure and arrival continents were identified and the average number of passengers calculated for every Monday between April 1 and May 31, which number was then applied to every other Monday in the year; values for other weekdays were similarly determined. For international mobility, we applied two hypothetical assumptions: (1) Individuals in the infected compartment do not travel across populations. This stems from the fact that infected individuals develop severe symptoms, such as a rash, which are easily identifiable and seriously affect their daily life; moreover, passengers with fever, which is one of the symptoms of MPX, would very likely be denied boarding during the post-COVID-19 period (56). Therefore, for each continent i, we set *I*_*ij*_ and *I*_*ji*_ to 0 (any j). (2) Since we are considering the early stage of the current pandemic, the number of recovered individuals that travel across populations is negligible and hence hardly affects transmission of the disease. Therefore, for each continent i, we set *R*_*ij*_ and *R*_*ji*_ to 0 (any j).

As a second improvement, we considered the age distribution of the MPX-susceptible population. It has been reported that for people vaccinated against smallpox, the chance of getting MPX would be 1/5.2 times that for those without a vaccination. Given that administration of the smallpox vaccine ceased by the 1980s, we assume that the population over 40 years old was vaccinated while the population under 40 years old was not (4, 59). Therefore, we adjusted the initial value (and maximum value as time changed) of susceptible individuals on continent i (*S*_*i*_) to


$$\begin{array}{lll} n_{i} & = & Population\;under\;40\;on\;continent\;i\;+\;\left(1/5.2\right)\;*\;population\\ above\;40\;on\;continent\;i \end{array}$$

Thirdly, the MPX outbreak on 23 June has caused global concern that could lead to changes in transmission rates. Accordingly, we fitted two phases, with the period before 23 June being termed the early stage of the epidemic and the period after 23 June being the current stage. For continent i, we denoted its transmission rate before 23 June as β_*i*1_, and that after 23 June as β_*i*2_. We then fitted β_*i*1_, β_*i*2_ and derived the epidemic curve by minimizing the mean square error (MSE). The resulting loss function is:


$$\begin{array}{ll} f\left(\beta_{i1},\beta_{i2}\right)\\ = & \sum_{i=1}^{6}\sum_{t=\;days\;from\;31\;Jan}^{16Aug}{\left(\frac{I_{i}\left(t\right)+R_{i}\left(t\right)-\;cumulative\;confirmed\;cases\;on\;continent\;i\left(t\right)}{\;cumulative\;confirmed\;cases\;on\;continent\;i\;\left(16\;Aug\right)}\right)}^{2} \end{array}$$

In terms of parameter selection, we took the median and set the incubation rate σ = 1/13 (reciprocal of the latent period) and the recovery rate γ = 1/21 (reciprocal of the recovery period); these values are based on a report that the latent period of MPX ranges from five to 21 days, while the recovery period is 2–4 weeks (31).

We assessed forecast errors using the normalized mean squared error (NMSE): the smaller the NMSE, the better the model fit. For continent j:


$$\begin{array}{l} NMSE_{j}=\frac{\overset{Aug\;16}{\underset{i=Jan\;31}{\sum }}{\left(c_{i}-\hat{c_{i}}\right)}^{2}}{\overset{Aug\;16}{\underset{i=Jan\;31}{\sum }}{c_{i}}^{2}}\; \end{array}$$

where *c*_*i*_= actual confirmed cases on date i j, $\hat{c_{i}}=$ predicted confirmed cases on date i of continent j.

### Statistical analysis

Descriptive analysis was performed using medians with interquartile ranges (IQR) for continuous variables and counts with percentages for categorical variables. When comparing differences between groups, the Kruskal-Wallis test and Wilcoxon test were used for continuous variables and the chi-square test for categorical variables.

## Results

### Spatiotemporal distribution of MPX cases

**Figure 1:**
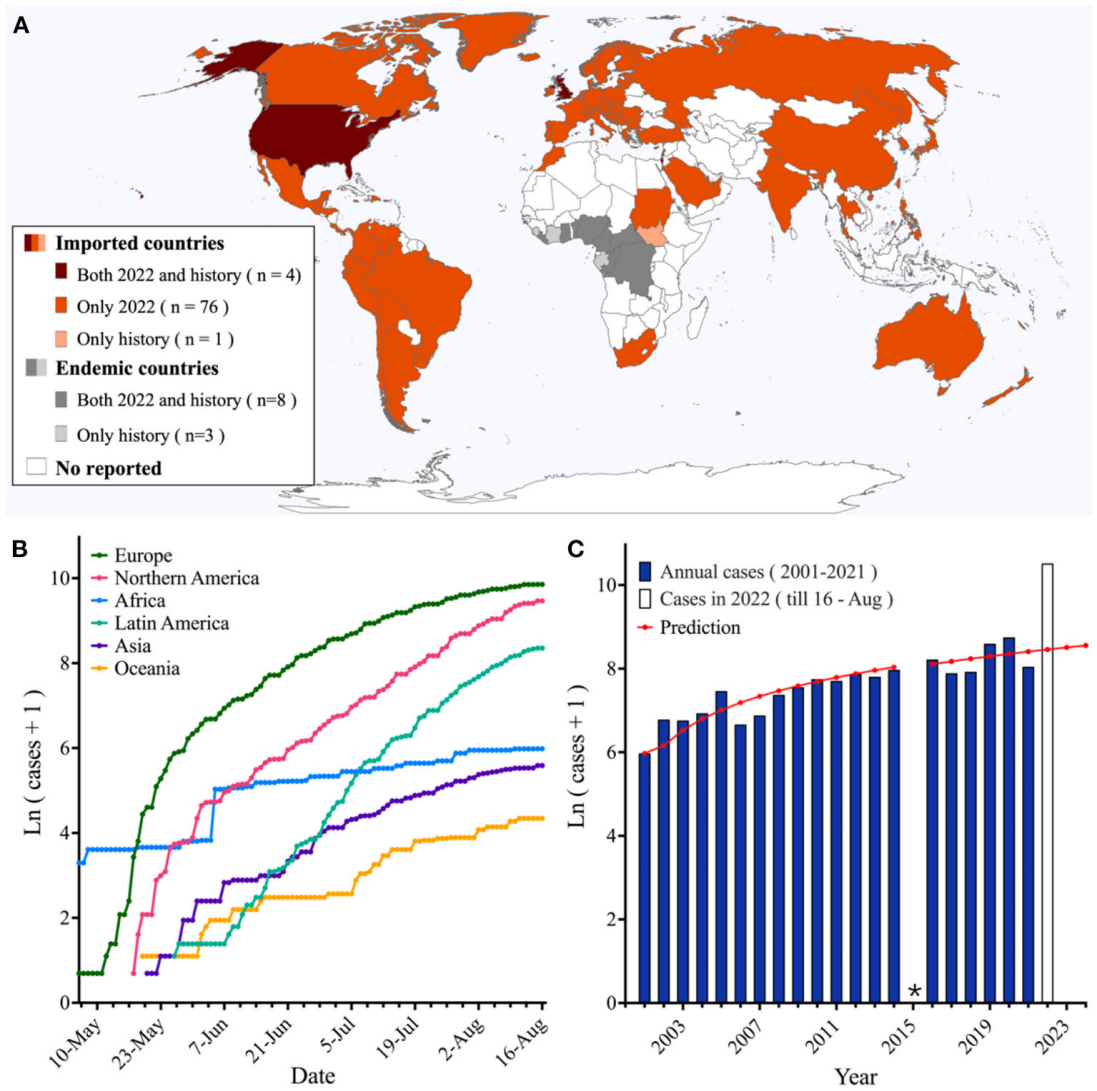
Spatiotemporal distribution of MPX infections. **(A)** Geographic distribution of reported MPX cases from 1970 to 2022. **(B)** Epidemiological curves of MPX in six continents from 6 May to 16 August 2022. **(C)** Prediction of yearly MPX case numbers from 2001 to 2024 using the GM (1, 1). *There is no prediction for 2015 due to a lack of observation data. Data obtained from the Global Health Team^1^ and the literature (23). MPX, monkeypox infections.

A total of 83,622 MPX cases were recorded worldwide since 2001, including 46,698 historical cases in 16 countries from 2001 to 2021, and 36,924 current epidemic cases in 88 countries as of 16 August 2022. In the current outbreak, 90.9% of infected countries were imported countries, with 86.4% reporting first infections, mostly in Europe (19,043 cases in 37 countries, 97.3% new-onset countries) and Northern America (12,904 cases in five countries, 75.0% new-onset countries) (Figures 1A, B). The dramatic increase in MPX cases observed as of 16 August 2022 was found to already exceed the GM (1, 1) forecast of 4,715 cases for the full year (mean squared difference ratio C value = 0.26, *p* = 0.80, Figure 1C).

### Main risk factors of the 2022 MPX outbreak

**Figure 2:**
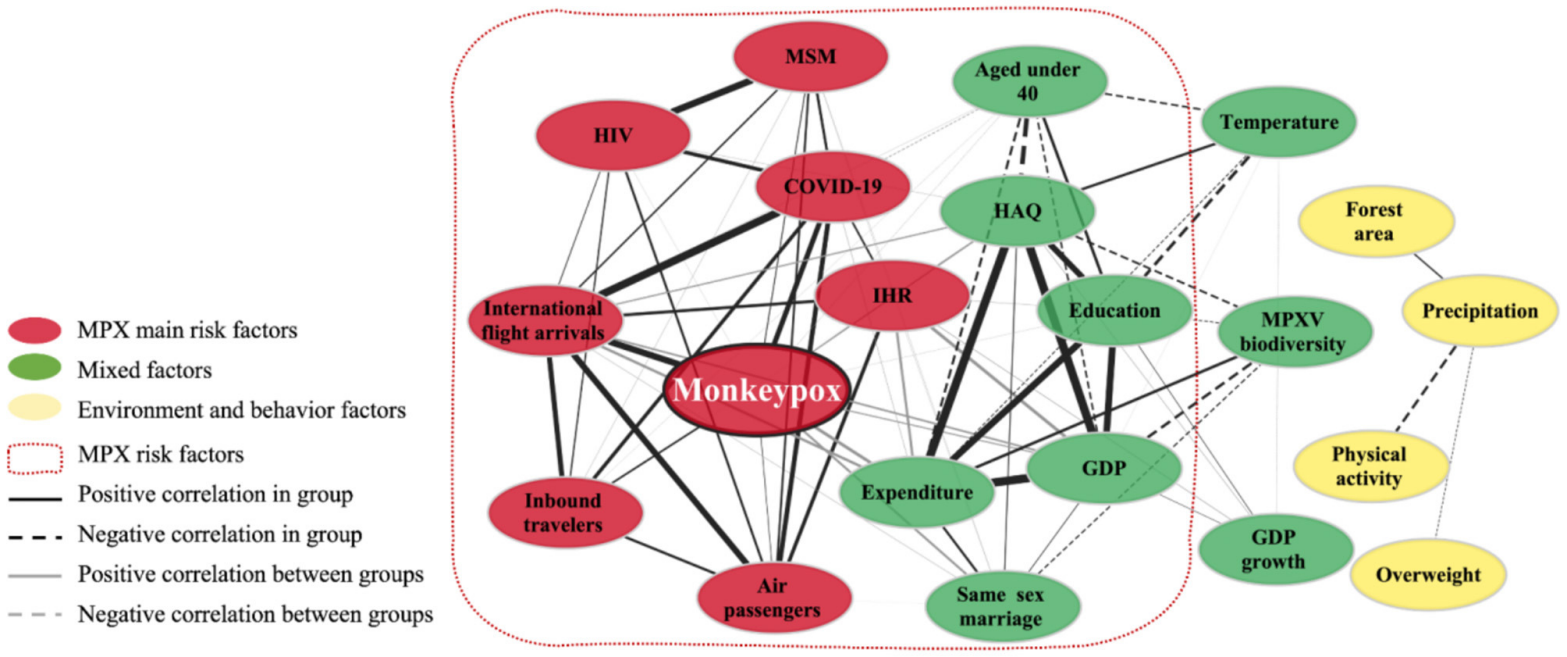
Risk factors associated with MPX infection in imported countries in 2022. Risk factors potentially affecting the 2022 outbreak were classified into three groups (colored ellipses). Red nodes are main risk factors. Green nodes are mixed factors. Yellow nodes are environment and behavior factors. Significant pairs identified by the Spearman correlation test (coefficient *r* < 0.3, *p* < 0.05) are connected by edges with the width representing the significance [–log10(*p*-value)]. MPX, monkeypox infections; GDP, gross domestic product per capita; MSM, men who have sex with men; MPXV, monkeypox virus; HIV, human immunodeficiency virus infections; COVID-19, coronavirus disease 2019 infections; HAQ, healthcare access and quality Index; IHR, international health regulations.

Risk factors influencing historical MPX infection were identified to be HIV infection and population density, while risk factors of the 2022 outbreak comprised multiple factors including human mobility, MSM population size, COVID-19 infection, and several socioeconomic factors (Figure 2; Supplementary Figure 4). Correlation analyses revealed 2022 MPX outbreaks in imported countries to involve 13 risk factors in the dimensions of socio-economic factors (GDP, legal same-sex marriage, MSM population size, and age), health burden (HIV infection and COVID-19 infection), health services (IHR, HAQ, and health expenditure), and mobility (international flight arrivals, air passengers, and inbound travelers) (Supplementary Table 5). Network analysis further highlighted seven main risk factors: international flight arrivals, COVID-19 infection, MSM population size, air passengers, HIV infection, IHR, and inbound travelers.

Finally, a multivariate negative binomial regression model showed that after adjusting for the interaction between international flight arrivals and COVID-19 infections, both of those parameters remained significantly associated with MPX infections (*p* < 0.001), while MSM population size, HIV infection and the IHR index did not (*p* > 0.05) (Table 2). For each additional one million national flight arrivals, the number of MPX cases increased up to 1.514 times (95% CI: 1.402–1.636). Countries with more COVID-19 infections also had more MPX infections, with each additional million COVID-19 cases corresponding to 1.286 times more MPX cases (95% CI: 1.223–1.353).

**Table 2:** Negative binomial regression analysis of association between number of MPX cases and key risk factors of MPX.

**Variable**	**Coefficient**	**Incidence rate ratio**	**95% CI**	***p*** **-value**
International flight arrivals (per million population)	0.415	1.514	1.402–1.636	<0.001
COVID-19 (per million population)	0.252	1.286	1.223–1.353	<0.001
Interaction effect between International flight arrivals and COVID-19	−0.015	0.985	0.982–0.988	<0.001

MPX, monkeypox infections; COVID-19, coronavirus disease 2019; CI, confidence interval.

### Global predictions using modified Flight-SEIR

**Figure 3:**
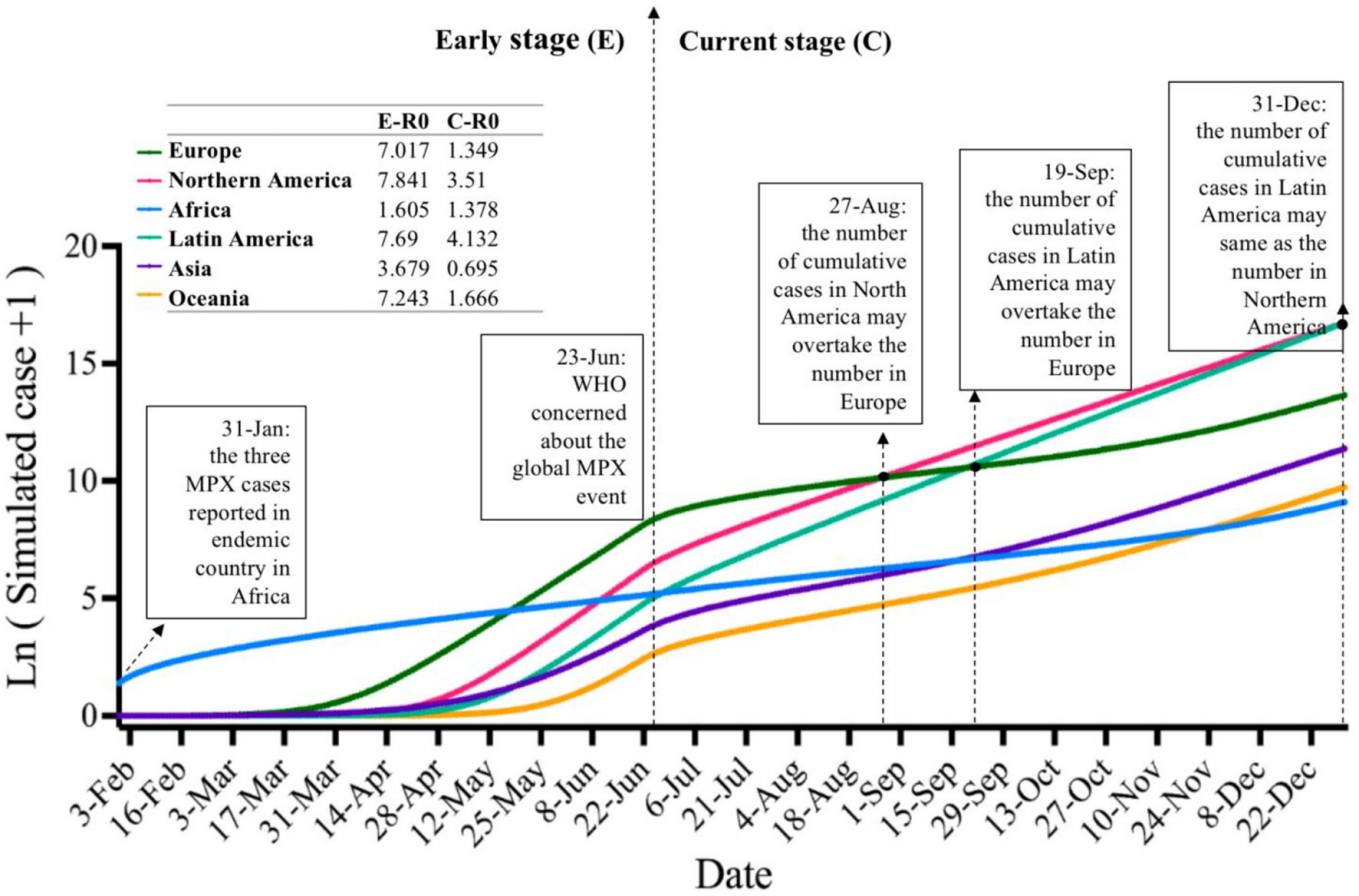
Simulated cumulative MPX cases on six continents predicted by a modified SEIR model. SEIR, susceptible-exposed-infectious-removed; MPX, monkeypox infections.

We constructed a Flight-SEIR model that took into account the human mobility associated with international flights and also simulated the gradual global attention to and managed transmission of MPX from 23 June onwards. This model's predictions matched well with the reported case data (NMSE ranging from 0.004 to 0.030) (Figure 3; Supplementary Figure 6). In the early stage (E stage) before 23 June 2022, the transmission rate was relatively high and infections surged on six continents. During this period, MPX spread most rapidly in Europe, Oceania, Northern America, and Latin America, with basic reproduction numbers (*R*_0_) ranging from 7.01 to 7.84; transmission rates were lower in Asia and Africa (*R*_0_ = 3.68 and 1.61, respectively). In the current stage (C stage) after 23 June, MPX transmission rates decreased significantly in all continents except Africa. The median *R*_0_ of medium-high-risk countries and region clusters declined from 7.01 to 1.35, while the *R*_0_ of the low-risk cluster was relatively stable, ranging 1.61–1.37. Geographically, *R*_0_ values remained relatively high in Latin America and Northern America (*R*_0_ = 4.13 and 3.51, respectively), moderate in Oceania and Europe (*R*_0_ = 1.67 and 1.35, respectively), and lowest in Asia (*R*_0_ = 0.70). While Europe currently has the highest cumulative cases, due to the continued rapid increase in cases in Northern America and Latin America, it is likely that these regions will overtake Europe by autumn 2022.

### Country-level risk classification for the MPX 2022 outbreak

**Figure 4:**
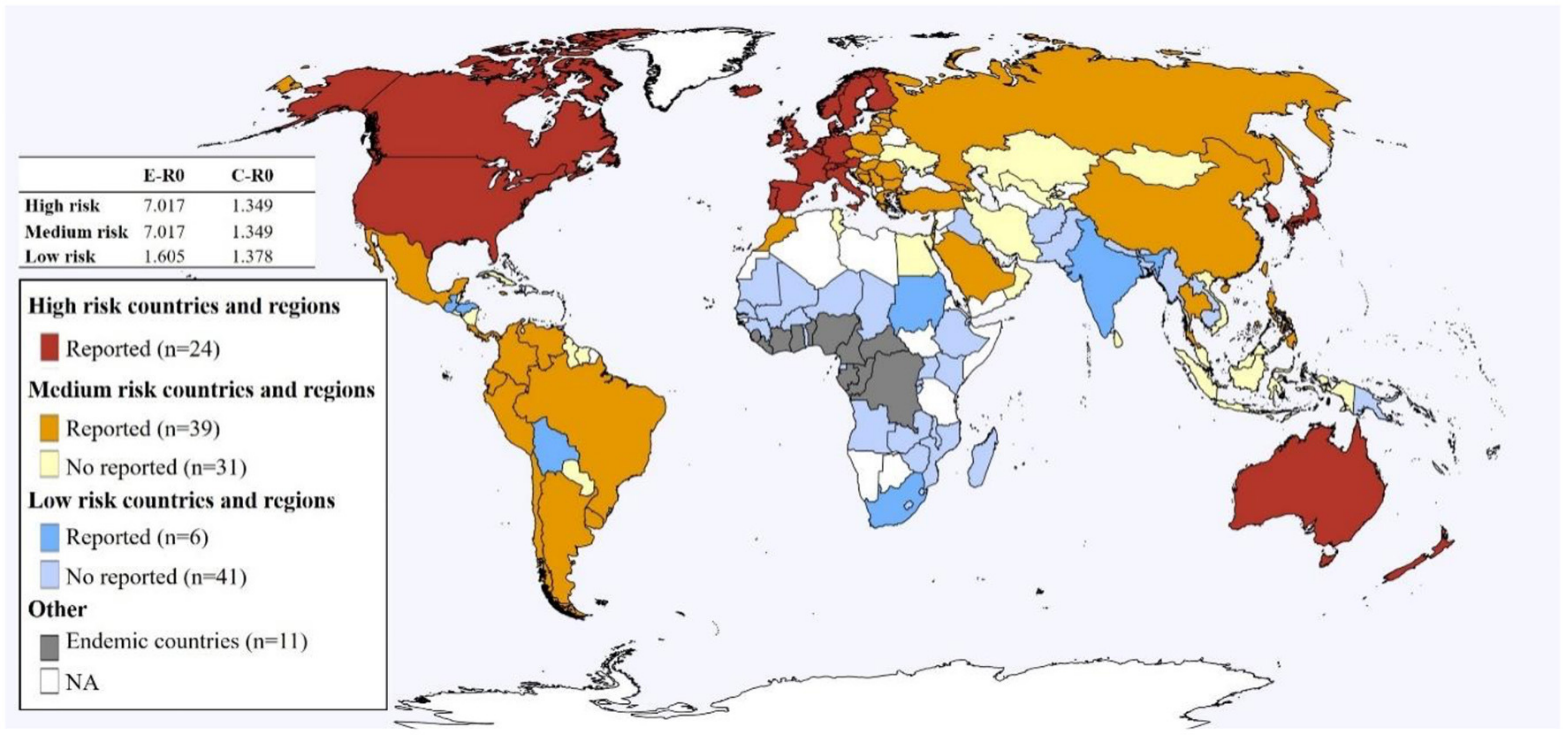
Country classification based on key risk factors.

Country-level risks around the globe were assessed by a k-means algorithm incorporating ten comprehensive risk factors. Of the 141 countries and regions examined, 56 countries were removed due to missing values and the remainder divided into three clusters (Figure 4; Supplementary Figure 3). Country risk level classifications were assigned based on MPX transmission and case count. The different clusters were further identified as being of high or low risk based on MPX cases and the proportion of affected countries (Figure 4). Countries and regions comprising the high-risk cluster (*n* = 24 countries, 31,639 cases, all member countries with reported MPX cases) were mainly located in West Europe and Northern America and featured high mobility, high socioeconomic level, COVID-19 infection, and a large MSM population, as well as a high *R*_0_ in the early stage of the outbreak (median 7.017) (Figure 4; Supplementary Figure 5). The low-risk cluster (*n* = 47, 36 cases, 12.8% of member countries with reported MPX cases) consisted mainly of countries located in Africa and South Asia, presented the opposite characteristics when compared to the high-risk cluster, and had a lower *R*_0_ in the early epidemic (median 1.605) (Figure 4; Supplementary Figure 5). Countries of the medium-risk cluster (*n* = 70, 4,723 cases, 55.7% of member countries with reported MPX cases) were mainly located in Latin America and Asia, and their characteristics were in-between the other two groups (Figure 4; Supplementary Figure 5).

## Discussion

This is the first study to assess global risks and predict epidemic trends for MPX. We comprehensively identify the risk factors that impact MPX infections and establish a country-level risk model and continent-level SEIR prediction model. We draw three conclusions from the findings of this work. Firstly, we showed for the first time that MPX risk factors have changed in this epidemic compared to the historical situation, and that multiple factors contribute to risk of re-emerging MPX infections. Our global country risk classification indicates that high-risk areas correspond to countries with more international flight arrivals, high socioeconomic level, and large MSM populations, and that are also characterized by high COVID-19 infections, health care capacity, and education level. Notably, our results highlight human mobility as a key risk factor in the current MPX outbreak; that is, MPX outbreaks in 2022 in non-endemic countries are often associated with international travel. Previous studies have attempted to elucidate the relationship between MPX transmission and air travel but were very limited (60). We found that an increase in international flight arrivals is significantly associated with MPX outbreaks on a global scale. As transport networks continue to expand and develop, global vulnerability to infectious diseases will be increased due to the greater likelihood of geographic transmission (61). Countries having less developed infrastructure or weaker capacity to respond to public health threats will face greater threat from infectious disease, and it is particularly important to improve their capacity to prevent, detect, and control epidemics.

Moreover, the difference in risk factors for the current global outbreak relative to the historical situation may have been influenced by the removal of restrictions related to COVID-19. To further clarify the role of COVID-19 in this outbreak, we compared historical MPX risk factors with those for 2022. From 2001 to 2021, MPX outbreaks were mainly associated with population density and HIV, and no significant correlation was found between MPX and COVID-19, even though COVID-19 had spread globally by 2020. In contrast, a significant univariate correlation was found between MPX and COVID-19 case counts in 2022. To adjust for confounding effects, we further constructed a multivariate regression model, which revealed a significant interaction of international flight arrivals with COVID-19 infection, with the univariate effect of COVID-19 infection still being significant. This suggests that in addition to influencing the spread of MPX through restriction policies that affect the number of international flight arrivals, COVID-19 infection may also affect it through other pathways such as potential changes in the psychological behavior and immune status of the population (10, 62, 63). After the world entered into the post-COVID-19 era around March 2022, most countries removed travel restrictions and gradually returned to their pre-pandemic lifestyles (16), which may have provided favorable circumstances for the emergence and spread of MPX. In addition, the overlap between areas of high COVID-19 prevalence and high MPX prevalence means that health systems will be under dual pressure and hidden danger of “combination virus”, and therefore the lessons learned from COVID-19 should be applied to develop control and prevention measures as soon as possible (7). Beyond COVID-19, MPX was also associated with HIV and with factors relating to epidemic response capacity such as IHR, HAQ, and health expenditure. Specifically, number of HIV infections was positively associated with number of MPX infections, and although these two viruses are not transmitted in exactly the same way, some countries are concerned about having a high prevalence of both infectious diseases. IHR, HAQ, and health expenditure were also positively associated with MPX infection, which may indicate good surveillance efforts in countries with high values for those factors.

The second conclusion of this study concerns the future trajectory of the current MPX outbreak. We assessed and predicted the epidemiological trends of MPX on a global scale using a Flight-SEIR model that accounted for human mobility, the age distribution of the susceptible population, and changes in transmission rate due to global concern. Our simulations using this model achieved a good fit, determined *R*_0_ by continent for the early and current stages of the epidemic, and predicted that Northern America and Latin America will have more MPX infections than Europe in the autumn of 2022. In the early stages of the outbreak, transmission rates were high on all continents except Africa and Asia, with one infected person being able to infect around seven people. Following the rise in global concern over MPX, transmission rates decreased significantly in Europe, Oceania and Asia, and declined but remained high in Latin America and Northern America. Transmission rates in Africa have been relatively stable throughout the current outbreak. The significant reduction of MPX transmission in Europe is likely due to the fact that Europe reported its first imported case of MPX half a month earlier than other continents, and on 2 June the European Center for Disease Prevention and Control (ECDC) issued interim recommendations on risk communication and community engagement (64), thereby providing timely and consistent health information and advice. Latin America, the last continent to report MPX cases, has been experiencing high rates of transmission and is likely to overtake Europe in late September, becoming the area with the highest cumulative number of cases by the end of the year if the spread continues at its current rate. The transmission rate of MPX remains above one in all continents except Asia, so the outbreak is still growing.

Thirdly, whereas MPX was once highly prevalent in endemic areas, most of the current cases had no history of staying in an infected area, and a high proportion involved MSM (65). Our study also showed a positive association between number of MPX infections and MSM population size at the national level. Although the start of the current MPX outbreak in this population may be coincidental, the risk of further transmission in the MSM population may be higher due to the prevalence of MPX in that population. Effective health education and community engagement among MSM are essential to protect this population as well as end MPX outbreaks. Stigmatization of MSM groups should be avoided in outbreak control and health promotion. In addition, many MSM with MPX are co-infected with other sexually transmitted diseases (STDs) (66), and comorbidities may pose multiple risks for serious health outcomes, so screening and treatment of STDs in the MSM population is also important. As the outbreak continues and the infected population expands, previously susceptible populations such as children are also at risk of MPX infection (23, 67); notably, pediatric MPX infection is associated with a higher risk of serious illness and mortality compared to adults (68). Therefore, infection control policies, contact tracing, and emergency vaccination of high-risk groups are necessary to prevent widespread transmission of MPX in the population.

There are several limitations in our analysis. First, our data were drawn from multiple open data sources. After comparison, we selected the more credible sources and applied outlier processing, but the credibility of the analysis relies heavily on the quality of the data. Second, as the world enters the post-COVID-19 era, COVID-19 infections are reported with limited accuracy and therefore may suffer from information bias. Third, our correlation analysis is based on country-level data rather than individual data, and sometimes reflects only the characteristics of countries without a causal association; as such, the results should be interpreted cautiously. Fourth, our SEIR model takes into account human mobility and the age structure of the population; if more data becomes available as the epidemic develops, more factors may need to be taken into account. Finally, we focused on the risks in countries to which MPX was imported and conducted only limited analysis on endemic countries, mainly due to the characteristics of the 2022 outbreak and there being insufficient data from such countries.

## Conclusion

Our study identifies variation in MPX infection across time and in different countries, and suggests the importance of conducting risk assessment studies supported by multidimensional factors. Forecasts of epidemiological trends from our modified SEIR models suggest that Northern America and Latin America are at greater risk of MPX infection in the future. Our global-scale study reminds policy makers that for prevention and control of MPX infection worldwide in the post-COVID-19 era, it is advisable to strengthen management in multiple dimensions such as human mobility, socio-economics, and sexual behavior.

## Data availability statement

The original contributions presented in the study are included in the article/Supplementary material, further inquiries can be directed to the corresponding authors.

## Author contributions

JG and CZho: data collection, investigation, data analysis, and writing—original draft and revision. HL: data collection, investigation, data curation, and writing. RJ: data analysis and writing. ÅMW and KJ: conceptualization. KJ and YG: investigation. JM and CZha: data collection and writing. SL: conceptualization and supervision. WL: conceptualization, supervision, and funding acquisition. LX: conceptualization, supervision, funding acquisition, writing, and revision. All authors contributed to the article and approved the submitted version.

## Footnotes

^1^The Global Health Team. Available from: https://github.com/globaldothealth/monkeypox (accessed August 17, 2022).

^2^*International Flight Arrivals*. Available from: https://uk.flightaware.com (accessed July 2, 2022).
