# Supplementary material for: Monkeypox outbreaks in the context of the COVID-19 pandemic: Network and clustering analyses of global risks and modified SEIR prediction of epidemic trends
Source: Front Public Health. 2023 Jan 24;11:1052946. doi: 10.3389/fpubh.2023.1052946 (PMC9902715; doi:10.3389/fpubh.2023.1052946)
Supplement: Supplementary file 2 [file Data_Sheet_1.DOCX]

Supplementary Material

**
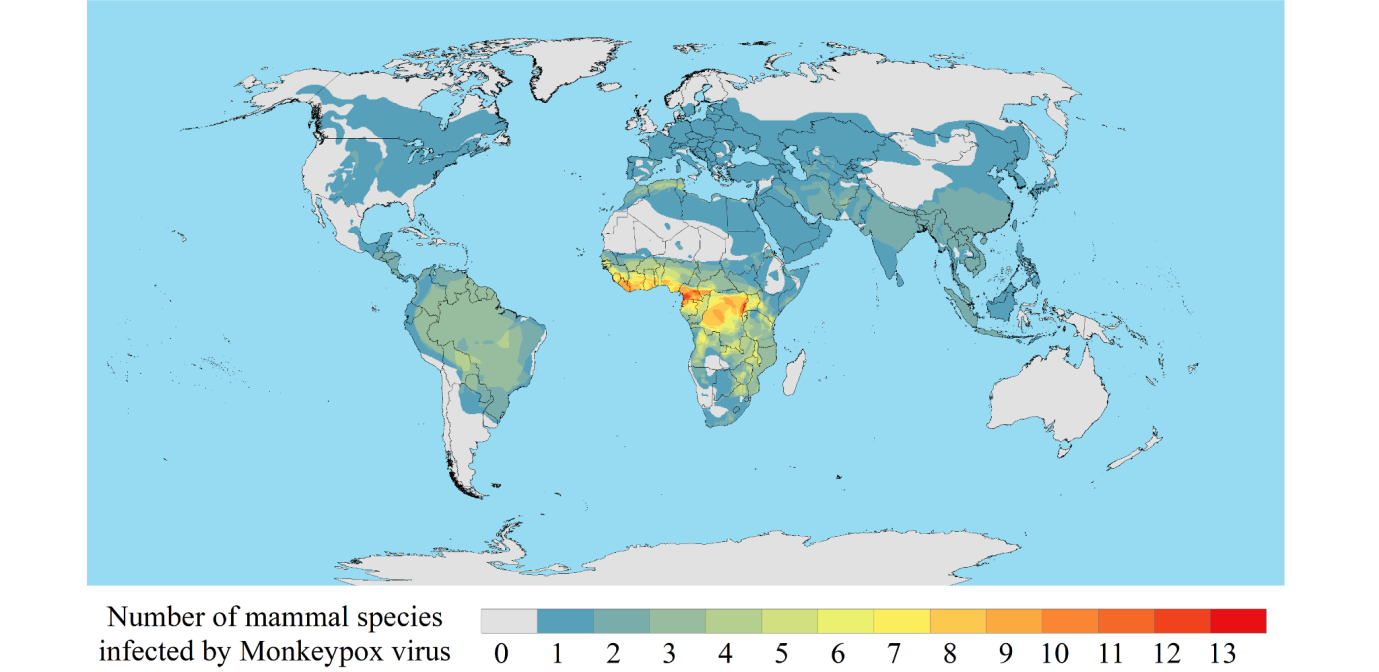
**

# Supplementary Figure 1. The spatial distribution of MPXV biodiversity worldwide

**
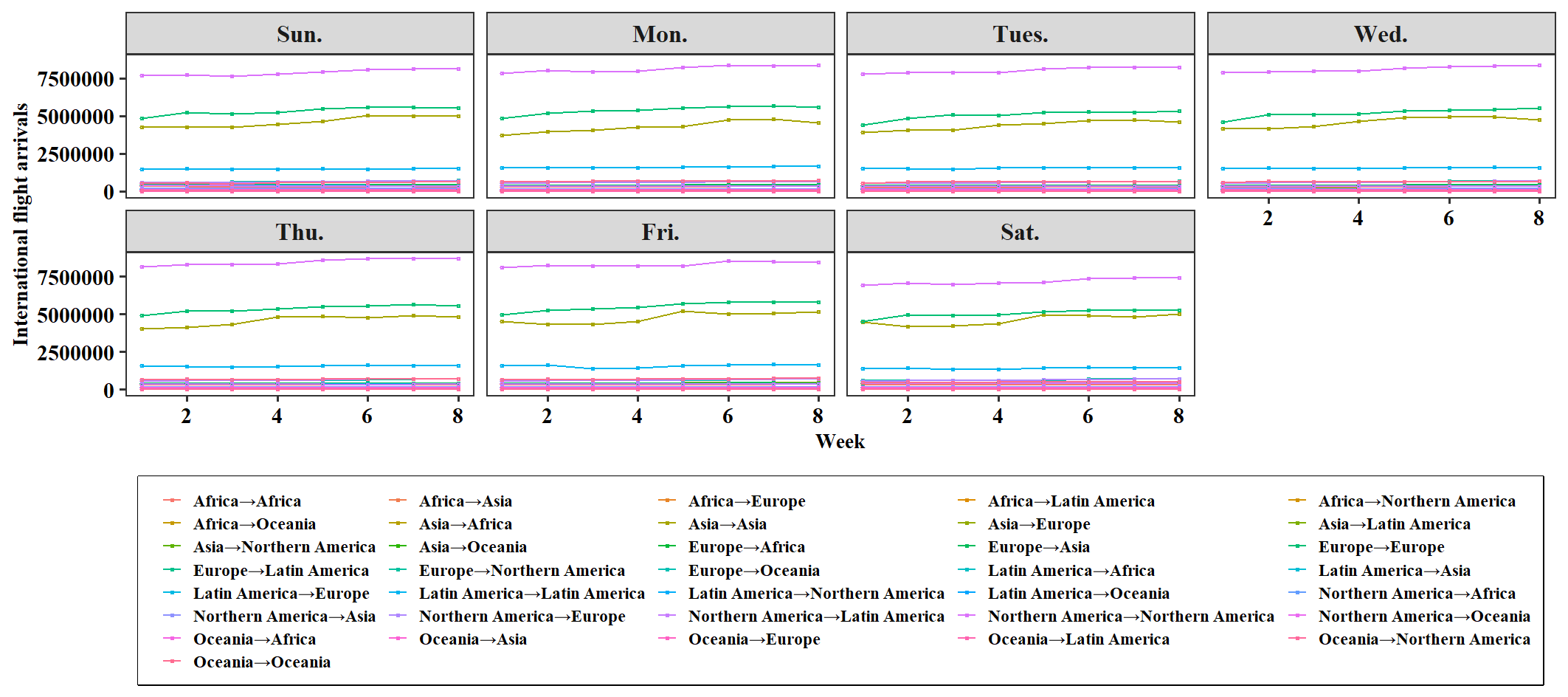
**

# Supplementary Figure 2. Global international flight arrivals status in April and May, 2022


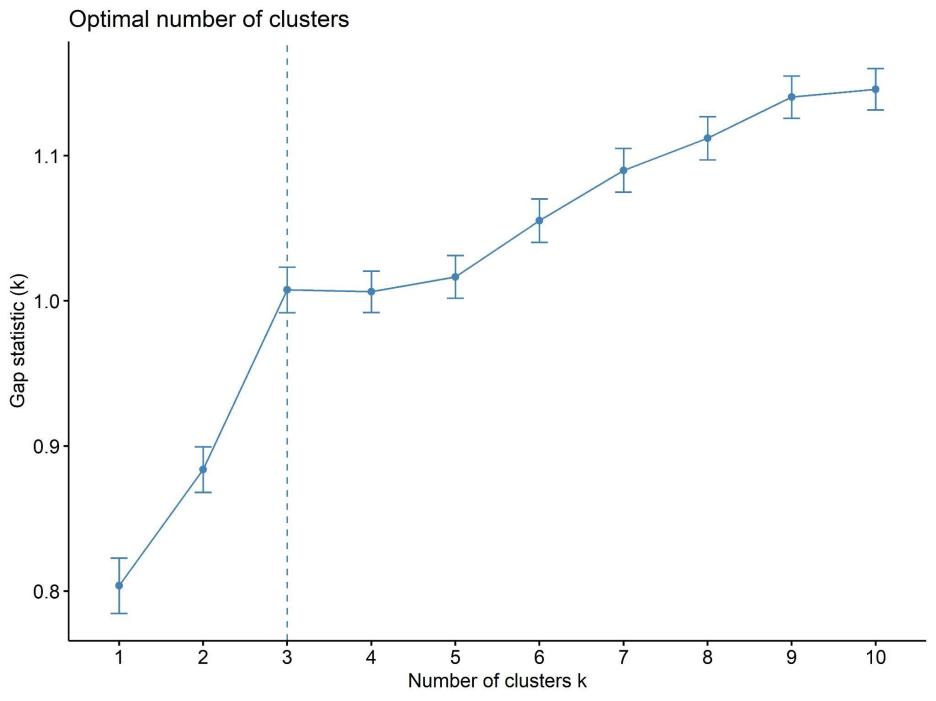


# Supplementary figure 3. Gap Statistic curve for k-means clustering to find optimal number of clusters


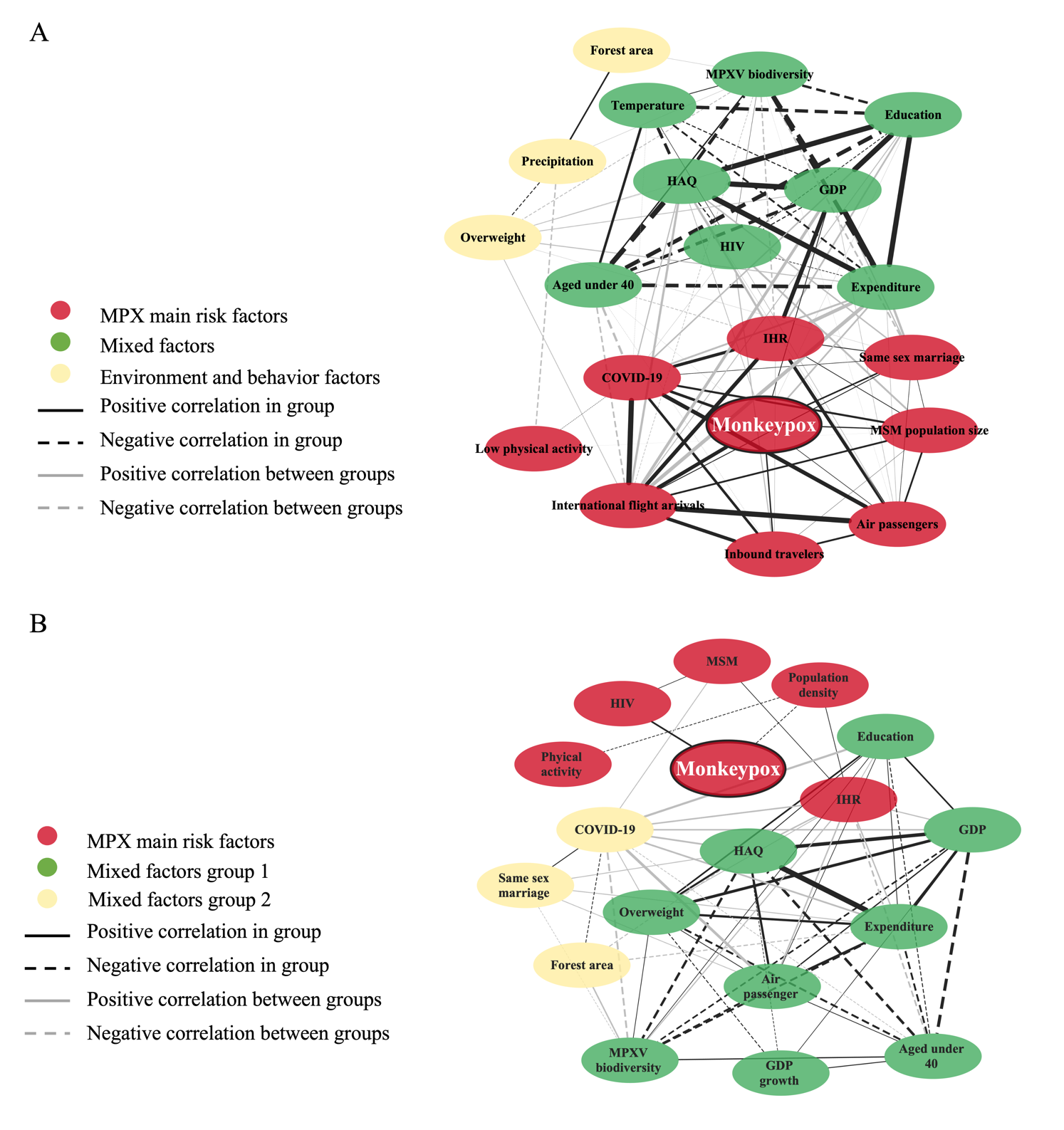


# Supplementary Figure 4. Assessment of risk factors of MPX infection

Note: (A) Risk factor assessment for all countries with MPX outbreaks in 2022. (B) Risk factor assessment for all countries with MPX outbreaks in history (2001-2021).


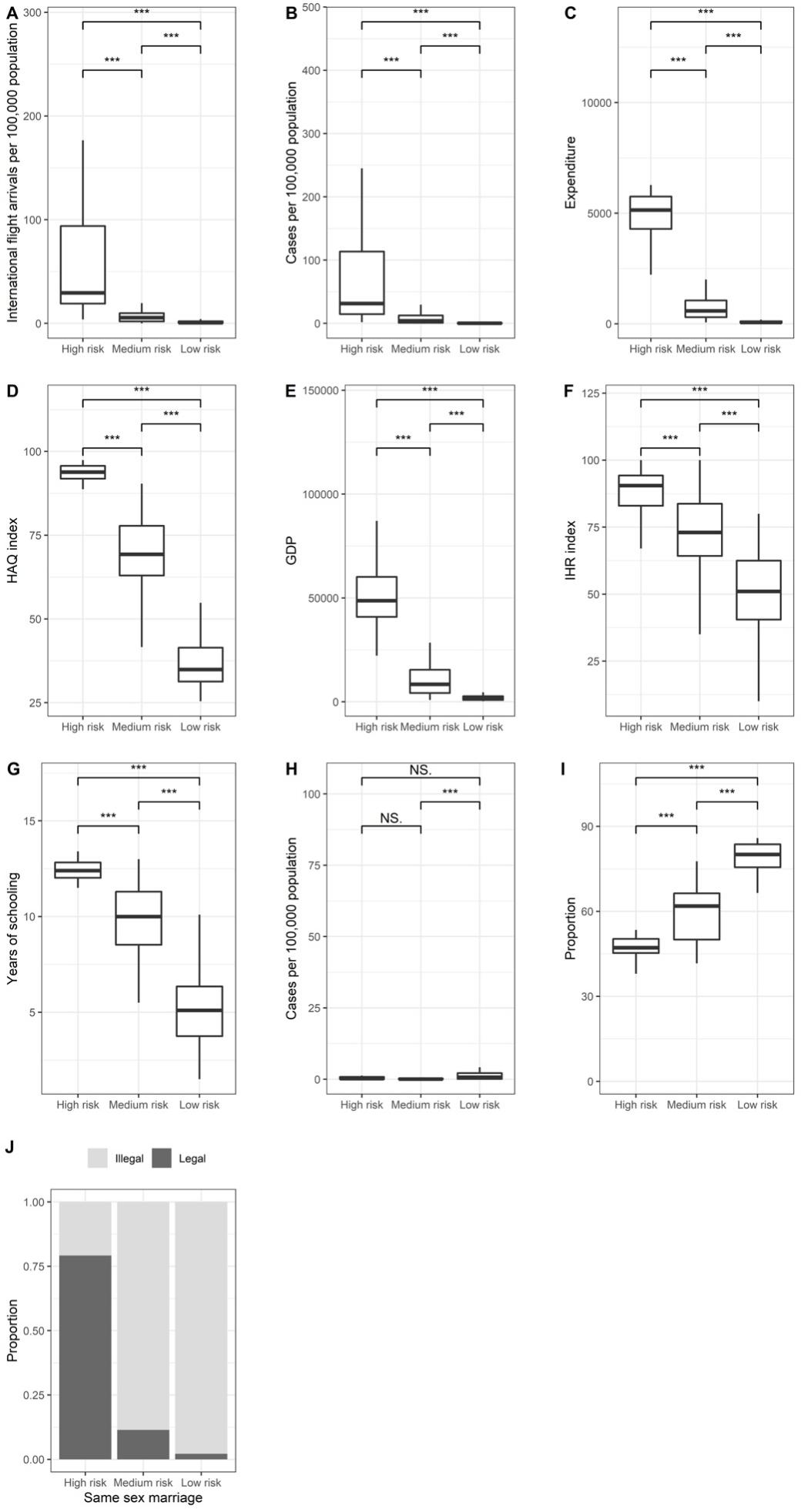


# Supplementary Figure 5. The levels of MPX risk factors between the three clusters.

Note: (A) international flight arrivals; (B) COVID-19 infections; (C) Expenditure; (D) HAQ index; (E) GDP; (F) IHR index; (G) Education; (H) HIV infections; (I) The proportion of population aged under 40 and (J) the proportion of legal same sex marriage

‘***’ for p-values < 0.001; ‘NS’ for p-values > 0.05 (no statistical significance). Abbreviations: MPX, monkeypox; GDP, gross domestic product; HIV, human immunodeficiency virus; COVID-19, coronavirus disease 2019; HAQ, healthcare access and quality; IHR, international health regulations.


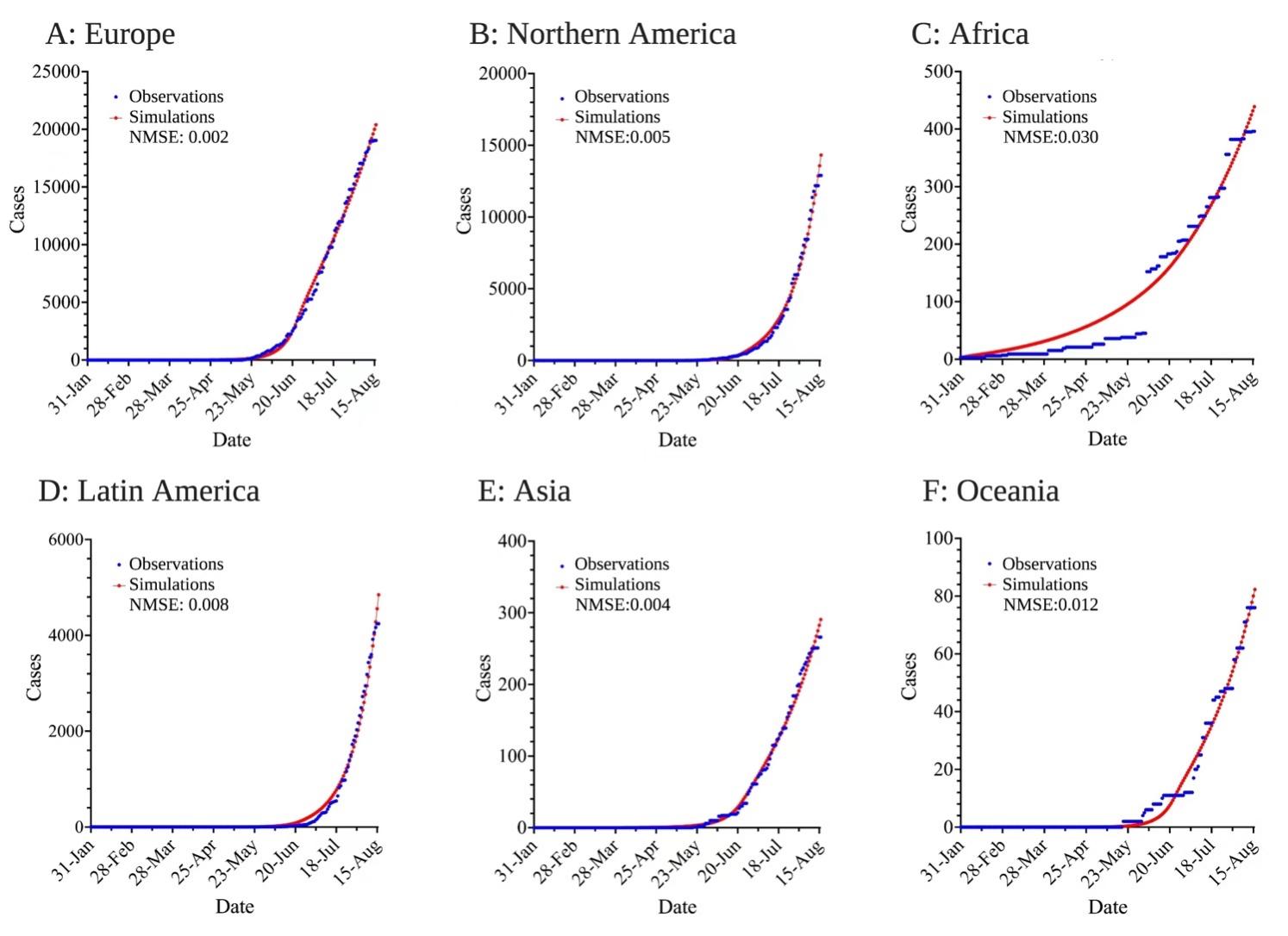


# Supplementary Figure 6. Simulations fitted by SEIR model for six continents.

Note: (A) Europe; (B) Northern America; (C) Africa; (D) Latin America; (E) Asia; (F) Oceania Abbreviations: NMSE, normalized mean square error

# Supplementary Table 1. Guidelines for Accurate and Transparent Health Estimates Reporting (GATHER)


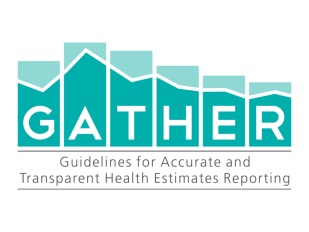


Checklist of information that should be included in new reports of global health estimates

| **Item #** | **Checklist item** | **Reported on page #** |
| --- | --- | --- |
| **Objectives and funding** | | |
| **1** | Define the indicator(s), populations (including age, sex, and geographic entities), and time period(s) for which estimates were made. | Main Text:  Methods. |
| **2** | List the funding sources for the work. | Main Text:  Acknowledgements and  declarations. |
| **Data Inputs** | | |
| **For all data inputs from multiple sources that are synthesized as part of the study:** | | |
| **3** | Describe how the data were identified and how the data were accessed. | Main Text:  Methods and table 1. |
| **4** | Specify the inclusion and exclusion criteria. Identify all ad-hoc exclusions. | Main Text:  Methods. |
| **5** | Provide information on all included data sources and their main characteristics. For each data source used, report reference information or contact name/institution, population represented, data collection method, year(s) of data collection, sex and age range, diagnostic criteria or measurement method, and sample size, as relevant. | Main Text:  Methods and table 1. |
| **6** | Identify and describe any categories of input data that have potentially important biases (e.g., based on characteristics listed in item 5). | Main text:  Limitations. |
| **For data inputs that contribute to the analysis but were not synthesized as part of the study:** | | |
| **7** | Describe and give sources for any other data inputs. | N/A |
| **For all data inputs:** | | |
| **8** | Provide all data inputs in a file format from which data can be efficiently extracted (e.g., a spreadsheet rather than a PDF), including all relevant meta-data listed in item 5. For any data inputs that cannot be shared because of ethical or legal reasons, such as third-party ownership, provide a contact name or the name of the institution that retains the right to the data. | Main Text:  Methods and table 1. |
| **Data analysis** | | |
| **9** | Provide a conceptual overview of the data analysis method. A diagram may be helpful. | Main Text:  Methods - overview. |
| **10** | Provide a detailed description of all steps of the analysis, including mathematical formulae. This description should cover, as relevant, data cleaning, data pre-processing, data adjustments and weighting of data sources, and mathematical or statistical model(s). | Main text:  Methods. |
| **11** | Describe how candidate models were evaluated and how the final model(s) were selected. | Main text:  Methods and Supplementary File 2 and 3 |
| **12** | Provide the results of an evaluation of model performance, if done, as well as the results of any relevant sensitivity analysis. | Supplementary Figure 6 |
| **13** | Describe methods for calculating uncertainty of the estimates. State which sources of uncertainty were, and were not, accounted for in the uncertainty analysis. | N/A |
| **14** | State how analytic or statistical source code used to generate estimates can be accessed. | N/A |
| **Results and Discussion** | | |
| **15** | Provide published estimates in a file format from which data can be efficiently extracted. | Main Text:  Methods and table 1. |
| **16** | Report a quantitative measure of the uncertainty of the estimates (e.g. uncertainty intervals). | N/A |
| **17** | Interpret results in light of existing evidence. If updating a previous set of estimates, describe the reasons for changes in estimates. | N/A |
| **18** | Discuss limitations of the estimates. Include a discussion of any modelling assumptions or data limitations that affect interpretation of the estimates. | Main text:  Limitations. |

# Supplementary Table 2. Distribution of countries with reported MPX cases worldwide

| MPX | Temporal coverage | Countries |
| --- | --- | --- |
| Endemic countries | History (n=11) | Africa: Benin; Cameroon; the Central African Republic; the Democratic; Republic of the Congo; Gabon; Ghana; Ivory Coast; Liberia; Nigeria; the Republic of the Congo; Sierra Leone |
|  | 2022 (n=8) | Africa: Benin; Cameroon; Central African Republic; Democratic Republic of the Congo; Ghana; Nigeria; Republic of Congo; Liberia |
| Imported countries | Only history (n=1) | Africa: South Sudan |
|  | History and 2022 (n=4) | Asia: Israel and Singapore  Europe: United Kingdom  Northern America: United States |
|  | Only 2022 (n=76) | Africa: South Africa; Morocco; Sudan  Asia: Georgia; Lebanon; South Korea; China (Taiwan); United Arab Emirates; Cyprus; India; Japan; Philippines; Qatar; Saudi Arabia; Thailand; Turkey  Europe: Austria; Belgium; Bulgaria; Croatia; Czech Republic; Denmark; Estonia; Finland; France; Germany; Gibraltar; Greece; Hungary; Iceland; Ireland; Italy; Latvia; Luxembourg; Malta; Netherlands; Norway; Poland; Portugal; Romania; Serbia; Slovenia; Spain; Sweden; Switzerland; Andorra; Bosnia and Herzegovi; Lithuania; Monaco; Moldova; Montenegro; Russian Federation; Slovak Republic.  Latin America: Argentina; Brazil; Chile; Colombia; Mexico; Peru; Venezuela;Bolivia;Barbados; Costa Rica; Dominican Republic; Ecuador; Guatemala; Honduras; Jamaica; Pama; Puerto Rico, Uruguay.  Northern America: Canada; Bermuda; Greenland  Oceania: Australia; New Caledonia; New Zealand |

MPX, monkeypox infection

# Supplementary Table 3. The list of variables used in analysis

| Variable | Description | 2001-2021 (All) | | | 2022 | | | | | Data source |
| --- | --- | --- | --- | --- | --- | --- | --- | --- | --- | --- |
|  |  | Temporal coverage | Value | Missing | Temporal coverage | Endemic | Missing | Imported | Missing |  |
| Population Density | Population density (people per sq. km of land area) | 2001-2020 | 46.4 [27.2-235] | 0 (0%) | 2020 | 56.2 [27.8-109] | 0 (0%) | 93.0 [31.8-220] | 0 (0%) | [https://data.worldbank.org/indicator/EN.POP.DNST (accessed 30 May 2022).](https://data.worldbank.org/indicator/EN.POP.DNST (accessed 30 May 2022)." \o "https://data.worldbank.org/indicator/EN.POP.DNST (accessed 30 May 2022).) |
| Aged under 40 | Proportion of 0- to 39-year olds in the general population | 2005-2020 | 80.0 [57.3-81.9] | 0 (0%) | 2020 | 81.7 [80.1-82.5] | 0 (0%) | 51.2 [46.0-63.7] | 6 (7.5%) | [https://www.un.org (accessed 30 May 2022).](https://www.un.org) |
| Education | Average number of years the population older than 25 participated in formal education | 2001-2017 | 5.93 [4.14-11.9] | 0 (0%) | 2017 | 6.20 [4.50-6.55] | 0 (0%) | 11.3 [9.25-12.4] | 5 (6.3%) | <https://ourworldindata.org/global-education (accessed 20 Jun 2022)> |
| GDP per capita | Per capita gross domestic product, a measure of a country's economic output per person | 2001-2020 | 2020 [526-39200] | 0 (0%) | 2020 | 1540 [588-2150] | 0 (0%) | 17700 [7680-43300] | 3 (3.8%) | [https://data.worldbank.org/indicator/NY.GDP.PCAP.CD (accessed 31 May 2022)](https://data.worldbank.org/indicator/NY.GDP.PCAP.CD (accessed 31 May 2022)" \o "https://data.worldbank.org/indicator/NY.GDP.PCAP.CD (accessed 31 May 2022)) |
| GDP growth (%) | Annual percentage growth rate of GDP at market prices based on constant local currency | 2001-2020 | -0.212 [-0.806-1.55] | 0 (0%) | 2020 | 0.414 [-1.90-1.28] | 0 (0%) | -5.23 [-7.78--2.95] | 0 (0%) | <https://data.worldbank.org/indicator/NY.GDP.MKTP.KD.ZG (accessed 31 May 2022).> |
| MSM population size (thousand)* | Estimated population of men who have sex with men | - | 85.8 [3.20-195] | 2 (18.2%) | - | 30.9 [3.85-70.0] | 1 (9.1%) | 88.0 [34.0-275] | 32 (40.0%) | <https://kpatlas.unaids.org/dashboard (accessed 7 Aug 2022).> |
| Same sex marriage | Legal status of same sex marriage | 2020 | legal 2 (18.2%) | 0 (0%) | 2020 | legal 0 | 0 (0%) | legal 31 (38.8%) | 0 (0%) | <https://ilga.org/maps-sexual-orientation-laws (accessed 6 Jun 2022).> |
| Forest area (%) | Proportion of land under natural or planted stands of trees of at least 5 meters in situ, whether productive or not; excludes tree stands in agricultural production systems | 2001-2020 | 33.8 [18.1-47.2] | 0 (0%) | 2020 | 35.8 [31.5-60.0] | 0 (0%) | 33.6 [18.6-47.2] | 2 (2.5%) | <https://data.worldbank.org/indicator/AG.LND.FRST.ZS (accessed 5 Jun 2022).> |
| Temperature | Mean temperature (degrees Celsius) | 2001-2020 | 26.1 [24.8-26.9] | 0 (0%) | 2020 | 26.4 [25.4-27.1] | 0 (0%) | 14.8 [10.1-24.4] | 3 (3.8%) | <https://climateknowledgeportal.worldbank.org/download-data (accessed 6 Jun 2022).> |
| Precipitation | Average precipitation (mm per year) | 2001-2020 | 1190 [850-1890] | 0 (0%) | 2020 | 1530 [1050-1870] | 0 (0%) | 900 [631-1390] | 3 (3.8%) | <https://climateknowledgeportal.worldbank.org/download-data (accessed 6 Jun 2022).> |
| MPXV | Diversity distribution of species that can be infected with MPXV | 2021 | 3.00 [0.500-6.00] | 0 (0%) | 2021 | 7.00 [5.00-7.00] | 0 (0%) | 1.00 [0-1.00] | 0 (0%) | <https://www.iucn.org/ (accessed 3 Jun 2022)> |
| HIV | The number of age-standardized HIV infections | 2001-2019 | 101000 [53300-329000] | 0 (0%) | 2019 | 104000 [72900-409000] | 0 (0%) | 12600 [1830-73600] | 2 (2.5%) | <https://vizhub.healthdata.org/gbd-results (accessed 7 Aug 2022).> |
| COVID-19 | The number of COVID-19 cases | - | 46200 [11100-536000] | 0 (0%) | 2022 Jan 1-Mar 31 | 5790 [2250-11100] | 0 (0%) | 839000 [277000-2820000] | 1 (1.3%) | <https://github.com/CSSEGISandData/COVID-19 (accessed 6 Jun 2022).> |
| HAQ | Healthcare Access and Quality Index | 2001-2016 | 48.5 [38.4-87.1] | 0 (0%) | 2016 | 32.2 [31.0-39.3] | 2 (18.2%) | 81.9 [68.5-91.9] | 4 (5.0%) | GBD 2016 Healthcare Access and Quality Collaborators |
| IHR | Average of 13 International Health Regulations core capacity scores, 1st version of the questionnaire | 2010-2020 | 55.7 [52.5-88.2] | 0 (0%) | 2020 | 49.0 [38.5-51.0] | 0 (0%) | 79.0 [67.0-88.3] | 4 (5.0%) | [https://www.who.int/data/gho/data/indicators/indicator-details/GHO/national-health-emergency-framework (accessed 31 May 2022).](https://www.who.int/data/gho/data/indicators/indicator-details/GHO/national-health-emergency-framework (accessed 31 May 2022)." \o "https://www.who.int/data/gho/data/indicators/indicator-details/GHO/national-health-emergency-framework (accessed 31 May 2022).) |
| Expenditure | Current health expenditure per capita in US$ | 2001-2019 | 69.2 [46.6-1940] | 0 (0%) | 2019 | 52.6 [41.7-73.3] | 0 (0%) | 1360 [545-3640] | 4 (5.0%) | [https://www.who.int/data/gho/data/indicators/indicator-details/GHO/current-health-expenditure-(che)-per-capita-in-us$ (accessed 31 May 2022)](https://www.who.int/data/gho/data/indicators/indicator-details/GHO/current-health-expenditure-(che)-per-capita-in-us$ (accessed 31 May 2022)" \o "https://www.who.int/data/gho/data/indicators/indicator-details/GHO/current-health-expenditure-(che)-per-capita-in-us$ (accessed 31 May 2022)) |
| Overweight | Prevalence of overweight among adults, BMI ≥ 25 (age-standardized estimate) | 2001-2016 | 26.7 [23.9-52.2] | 1 (9.1%) | 2016 | 30.9 [28.3-31.8] | 0 (0%) | 58.4 [55.8-62.3] | 4 (5.0%) | [https://www.who.int/data/gho/data/indicators/indicator-details/GHO/prevalence-of-overweight-among-adults-bmi-=-25-(age-standardized-estimate (accessed 7 Jul 2022).](https://www.who.int/data/gho/data/indicators/indicator-details/GHO/prevalence-of-overweight-among-adults-bmi-=-25-(age-standardized-estimate (accessed 7 Jul 2022)." \o "https://www.who.int/data/gho/data/indicators/indicator-details/GHO/prevalence-of-overweight-among-adults-bmi-=-25-(age-standardized-estimate (accessed 7 Jul 2022).) |
| Low physical activity | Disability-adjusted life years attributed to low physical activity | 2001-2019 | 203 [165-242] | 0 (0%) | 2019 | 208 [179-239] | 0 (0%) | 165 [131-238] | 2 (2.5%) | [https://www.who.int/data/gho/data/indicators/indicator-details/GHO/prevalence-of-overweight-among-adults-bmi-=-25-(age-standardized-estimate) (accessed 9 Jul 2022).](https://www.who.int/data/gho/data/indicators/indicator-details/GHO/prevalence-of-overweight-among-adults-bmi-=-25-(age-standardized-estimate) (accessed 9 Jul 2022)." \o "https://www.who.int/data/gho/data/indicators/indicator-details/GHO/prevalence-of-overweight-among-adults-bmi-=-25-(age-standardized-estimate) (accessed 9 Jul 2022).) |
| Inbound travelers | The number of tourists who travel to a country other than that in which they have their usual residence, but outside their usual environment, for a period not exceeding 12 months and whose main purpose in visiting is other than an activity remunerated from within the country visited. | 2001-2020 | 21900000 [14700000-58100000] | 8 (72.7%) | 2020 | 668000 [668000-668000] | 10 (90.9%) | 2520000 [807000-7340000] | 21 (26.3%) | [https://data.worldbank.org.cn/indicator/ST.INT.ARVL (accessed 2 Jun 2022).](https://data.worldbank.org.cn/indicator/ST.INT.ARVL (accessed 2 Jun 2022)." \o "https://data.worldbank.org.cn/indicator/ST.INT.ARVL (accessed 2 Jun 2022).) |
| Air passengers | Registered carrier departures worldwide; the domestic take offs and take offs abroad of air carriers registered in the country. | 2001-2020 | 3260000 [441000-30600000] | 3 (27.3%) | 2020 | 300000 [239000-322000] | 5 (45.5%) | 6220000 [795000-25000000] | 11 (13.8%) | https://data.worldbank.org.cn/indicator/IS.AIR.PSGR (accessed 3 Jun 2022). |
| International flight arrivals* | Worldwide flight traffic in April and May 2022. All data were filtered for routes across countries then aggregated from the airport level to the national level. | - | - | - | - | 60100 [36800-132000] | 0 (0%) | 968000 [391000-2490000] | 4 (5.0%) | [https://uk.flightaware.com (accessed 2 Jul 2022)](https://uk.flightaware.com) |

MPX, monkeypox; GDP, gross domestic product; MSM, men who have sex with men; MPXV, monkeypox virus; HIV, Human Immunodeficiency Virus; COVID-19, coronavirus disease 2019; HAQ, Healthcare Access and Quality; IHR, International Health Regulations; BMI, Body mass index; WHO, World Health Organization; GBD, Global Burden of Disease. *International flight arrivals: We collected real-time global flight traffic for April and May 2022, extracting route passengers by each continent on a daily basis. To fill in the gaps, we defaulted to a weekly cycle of flight data, so we averaged the seven days from Monday to Sunday for the eight weeks from April 1, 2022 to May 26, 2022, and repeated these seven days for each day from December 1, 2021 to June 30, 2022, with April 1, 2022 to May 31, 2022 as the real data

# Supplementary Table 4. Mammal species that were found to be infectious to the Monkeypox virus

| No | Species | Kingdom | Phylum | Class | Order | Family | Genus |
| --- | --- | --- | --- | --- | --- | --- | --- |
| 1 | Nasua nasua | Animalia | Chordata | Mammalia | Carnivora | Procyonidae | Nasua |
| 2 | Sus scrofa | Animalia | Chordata | Mammalia | Cetartiodactyla | Suidae | Sus |
| 3 | Didelphis marsupialis | Animalia | Chordata | Mammalia | Didelphimorphia | Didelphidae | Didelphis |
| 4 | Monodelphis domestica | Animalia | Chordata | Mammalia | Didelphimorphia | Didelphidae | Monodelphis |
| 5 | Atelerix albiventris | Animalia | Chordata | Mammalia | Eulipotyphla | Erinaceidae | Atelerix |
| 6 | Atelerix algirus | Animalia | Chordata | Mammalia | Eulipotyphla | Erinaceidae | Atelerix |
| 7 | Atelerix frontalis | Animalia | Chordata | Mammalia | Eulipotyphla | Erinaceidae | Atelerix |
| 8 | Atelerix sclateri | Animalia | Chordata | Mammalia | Eulipotyphla | Erinaceidae | Atelerix |
| 9 | Crocidura littoralis | Animalia | Chordata | Mammalia | Eulipotyphla | Soricidae | Crocidura |
| 10 | Petrodromus tetradactylus | Animalia | Chordata | Mammalia | Macroscelidea | Macroscelididae | Petrodromus |
| 11 | Myrmecophaga tridactyla | Animalia | Chordata | Mammalia | Pilosa | Myrmecophagidae | Myrmecophaga |
| 12 | Cercocebus atys | Animalia | Chordata | Mammalia | Primates | Cercopithecidae | Cercocebus |
| 13 | Macaca fascicularis | Animalia | Chordata | Mammalia | Primates | Cercopithecidae | Macaca |
| 14 | Macaca mulatta | Animalia | Chordata | Mammalia | Primates | Cercopithecidae | Macaca |
| 15 | Jaculus blanfordi | Animalia | Chordata | Mammalia | Rodentia | Dipodidae | Jaculus |
| 16 | Jaculus jaculus | Animalia | Chordata | Mammalia | Rodentia | Dipodidae | Jaculus |
| 17 | Jaculus orientalis | Animalia | Chordata | Mammalia | Rodentia | Dipodidae | Jaculus |
| 18 | Jaculus thaleri | Animalia | Chordata | Mammalia | Rodentia | Dipodidae | Jaculus |
| 19 | Graphiurus angolensis | Animalia | Chordata | Mammalia | Rodentia | Gliridae | Graphiurus |
| 20 | Graphiurus christyi | Animalia | Chordata | Mammalia | Rodentia | Gliridae | Graphiurus |
| 21 | Graphiurus crassicaudatus | Animalia | Chordata | Mammalia | Rodentia | Gliridae | Graphiurus |
| 22 | Graphiurus johnstoni | Animalia | Chordata | Mammalia | Rodentia | Gliridae | Graphiurus |
| 23 | Graphiurus kelleni | Animalia | Chordata | Mammalia | Rodentia | Gliridae | Graphiurus |
| 24 | Graphiurus lorraineus | Animalia | Chordata | Mammalia | Rodentia | Gliridae | Graphiurus |
| 25 | Graphiurus microtis | Animalia | Chordata | Mammalia | Rodentia | Gliridae | Graphiurus |
| 26 | Graphiurus monardi | Animalia | Chordata | Mammalia | Rodentia | Gliridae | Graphiurus |
| 27 | Graphiurus murinus | Animalia | Chordata | Mammalia | Rodentia | Gliridae | Graphiurus |
| 28 | Graphiurus nagtglasii | Animalia | Chordata | Mammalia | Rodentia | Gliridae | Graphiurus |
| 29 | Graphiurus ocularis | Animalia | Chordata | Mammalia | Rodentia | Gliridae | Graphiurus |
| 30 | Graphiurus platyops | Animalia | Chordata | Mammalia | Rodentia | Gliridae | Graphiurus |
| 31 | Graphiurus rupicola | Animalia | Chordata | Mammalia | Rodentia | Gliridae | Graphiurus |
| 32 | Graphiurus surdus | Animalia | Chordata | Mammalia | Rodentia | Gliridae | Graphiurus |
| 33 | Graphiurus walterverheyeni | Animalia | Chordata | Mammalia | Rodentia | Gliridae | Graphiurus |
| 34 | Atherurus africanus | Animalia | Chordata | Mammalia | Rodentia | Hystricidae | Atherurus |
| 35 | Cricetomys ansorgei | Animalia | Chordata | Mammalia | Rodentia | Nesomyidae | Cricetomys |
| 36 | Cricetomys emini | Animalia | Chordata | Mammalia | Rodentia | Nesomyidae | Cricetomys |
| 37 | Cricetomys gambianus | Animalia | Chordata | Mammalia | Rodentia | Nesomyidae | Cricetomys |
| 38 | Cynomys gunnisoni | Animalia | Chordata | Mammalia | Rodentia | Sciuridae | Cynomys |
| 39 | Cynomys leucurus | Animalia | Chordata | Mammalia | Rodentia | Sciuridae | Cynomys |
| 40 | Cynomys ludovicianus | Animalia | Chordata | Mammalia | Rodentia | Sciuridae | Cynomys |
| 41 | Cynomys mexicanus | Animalia | Chordata | Mammalia | Rodentia | Sciuridae | Cynomys |
| 42 | Cynomys parvidens | Animalia | Chordata | Mammalia | Rodentia | Sciuridae | Cynomys |
| 43 | Funisciurus anerythrus | Animalia | Chordata | Mammalia | Rodentia | Sciuridae | Funisciurus |
| 44 | Funisciurus bayonii | Animalia | Chordata | Mammalia | Rodentia | Sciuridae | Funisciurus |
| 45 | Funisciurus carruthersi | Animalia | Chordata | Mammalia | Rodentia | Sciuridae | Funisciurus |
| 46 | Funisciurus congicus | Animalia | Chordata | Mammalia | Rodentia | Sciuridae | Funisciurus |
| 47 | Funisciurus duchaillui | Animalia | Chordata | Mammalia | Rodentia | Sciuridae | Funisciurus |
| 48 | Funisciurus isabella | Animalia | Chordata | Mammalia | Rodentia | Sciuridae | Funisciurus |
| 49 | Funisciurus lemniscatus | Animalia | Chordata | Mammalia | Rodentia | Sciuridae | Funisciurus |
| 50 | Funisciurus leucogenys | Animalia | Chordata | Mammalia | Rodentia | Sciuridae | Funisciurus |
| 51 | Funisciurus pyrropus | Animalia | Chordata | Mammalia | Rodentia | Sciuridae | Funisciurus |
| 52 | Funisciurus substriatus | Animalia | Chordata | Mammalia | Rodentia | Sciuridae | Funisciurus |
| 53 | Heliosciurus gambianus | Animalia | Chordata | Mammalia | Rodentia | Sciuridae | Heliosciurus |
| 54 | Heliosciurus mutabilis | Animalia | Chordata | Mammalia | Rodentia | Sciuridae | Heliosciurus |
| 55 | Heliosciurus punctatus | Animalia | Chordata | Mammalia | Rodentia | Sciuridae | Heliosciurus |
| 56 | Heliosciurus rufobrachium | Animalia | Chordata | Mammalia | Rodentia | Sciuridae | Heliosciurus |
| 57 | Heliosciurus ruwenzorii | Animalia | Chordata | Mammalia | Rodentia | Sciuridae | Heliosciurus |
| 58 | Heliosciurus undulatus | Animalia | Chordata | Mammalia | Rodentia | Sciuridae | Heliosciurus |
| 59 | Marmota monax | Animalia | Chordata | Mammalia | Rodentia | Sciuridae | Marmota |

# Supplementary Table 5. Assessment of risk factors of MPX infection

Note: (A) in imported countries in 2022; (B) all infected countries in 2022, and (C) all infected countries in history

| A: Variable in imported countries in 2022 | | Weight | Correlation | P value |
| --- | --- | --- | --- | --- |
| Monkeypox | International flight arrivals | 11.89619628 | 0.704224103 | 1.27E-12 |
| Monkeypox | COVID-19 | 9.91721463 | 0.646698306 | 1.21E-10 |
| Monkeypox | Expenditure | 6.821023053 | 0.559309691 | 1.51E-07 |
| Monkeypox | Inbound travelers | 4.876148359 | 0.53398065 | 1.33E-05 |
| Monkeypox | MSM population size | 3.59667697 | 0.504822971 | 0.000253118 |
| Monkeypox | Same sex marriage | 5.473660723 | 0.493172684 | 3.36E-06 |
| Monkeypox | HAQ | 4.167491087 | 0.440584389 | 6.80E-05 |
| Monkeypox | GDP | 4.030584088 | 0.430468316 | 9.32E-05 |
| Monkeypox | Air passengers | 3.094165817 | 0.394154464 | 0.000805071 |
| Monkeypox | IHR | 2.343933916 | 0.322228989 | 0.004529665 |
| Monkeypox | Education | 2.308245387 | 0.321477159 | 0.004917616 |
| Monkeypox | HIV | 2.140957582 | 0.301887654 | 0.007228404 |
| Monkeypox | Aged under 40 | 2.511668037 | -0.339564244 | 0.003078449 |
| Aged under 40 | Temperature | 4.869666232 | 0.491448692 | 1.35E-05 |
| Aged under 40 | International flight arrivals | 2.351497037 | -0.331468262 | 0.004451465 |
| Aged under 40 | Inbound travelers | 2.389108275 | -0.381240981 | 0.004082176 |
| Aged under 40 | COVID-19 | 3.256010738 | -0.394421819 | 0.000554612 |
| Aged under 40 | Expenditure | 5.709965389 | -0.527300791 | 1.95E-06 |
| Aged under 40 | Education | 6.458420756 | -0.558273863 | 3.48E-07 |
| Aged under 40 | HAQ | 9.067526235 | -0.646508824 | 8.56E-10 |
| Air passengers | COVID-19 | 9.322393047 | 0.664584019 | 4.76E-10 |
| Air passengers | Inbound travelers | 5.991399828 | 0.623585847 | 1.02E-06 |
| Education | Expenditure | 12.47755577 | 0.720034775 | 3.33E-13 |
| Expenditure | COVID-19 | 2.663763875 | 0.346482204 | 0.002168883 |
| Forest area | Precipitation | 4.341988603 | 0.452660028 | 4.55E-05 |
| GDP | Expenditure | 15 | 0.954089289 | 0 |
| GDP | HAQ | 15 | 0.897749187 | 0 |
| GDP | Education | 12.40782324 | 0.72197577 | 3.91E-13 |
| GDP | IHR | 6.924453039 | 0.566323273 | 1.19E-07 |
| GDP | International flight arrivals | 4.935542011 | 0.485642355 | 1.16E-05 |
| GDP | GDP growth | 2.779016357 | 0.352542195 | 0.00166335 |
| GDP | Temperature | 2.140815693 | -0.309796372 | 0.007230766 |
| GDP | Aged under 40 | 4.812479279 | -0.482444774 | 1.54E-05 |
| GDP | MPXV biodiversity | 6.279840697 | -0.535380548 | 5.25E-07 |
| GDP growth | Education | 3.075172505 | 0.37751344 | 0.000841061 |
| GDP growth | IHR | 2.933787789 | 0.365576152 | 0.001164695 |
| GDP growth | HAQ | 2.356466098 | 0.323216374 | 0.004400823 |
| GDP growth | Temperature | 2.291448831 | -0.316028156 | 0.005111533 |
| HAQ | Expenditure | 15 | 0.91574598 | 0 |
| HAQ | Education | 11.25103714 | 0.696387209 | 5.61E-12 |
| HAQ | International flight arrivals | 4.269217724 | 0.454434658 | 5.38E-05 |
| HAQ | IHR | 3.712798638 | 0.420178022 | 0.000193732 |
| HAQ | HIV | 2.464982118 | -0.335972039 | 0.003427819 |
| HAQ | MPXV biodiversity | 5.156144577 | -0.490161987 | 6.98E-06 |
| HIV | MSM population size | 12.4710833 | 0.833950046 | 3.38E-13 |
| HIV | COVID-19 | 7.835647144 | 0.591618907 | 1.46E-08 |
| HIV | Air passengers | 5.568636236 | 0.534280043 | 2.70E-06 |
| HIV | Inbound travelers | 4.240332155 | 0.506805808 | 5.75E-05 |
| IHR | Air passengers | 7.723538196 | 0.614964276 | 1.89E-08 |
| IHR | Expenditure | 5.838631998 | 0.520383319 | 1.45E-06 |
| IHR | COVID-19 | 4.91721463 | 0.47888178 | 1.21E-05 |
| IHR | MSM population size | 2.183718521 | 0.387203739 | 0.006550606 |
| IHR | Inbound travelers | 1.997988713 | 0.34129667 | 0.010046419 |
| IHR | Education | 2.546963125 | 0.340027483 | 0.00283816 |
| Inbound travelers | COVID-19 | 7.669586227 | 0.656956535 | 2.14E-08 |
| International flight arrivals | COVID-19 | 14.1481304 | 0.752261735 | 7.11E-15 |
| International flight arrivals | Inbound travelers | 9.756961951 | 0.734415584 | 1.75E-10 |
| International flight arrivals | Air passengers | 11.61439373 | 0.729895838 | 2.43E-12 |
| International flight arrivals | MSM population size | 4.673664139 | 0.582978723 | 2.12E-05 |
| International flight arrivals | IHR | 6.630784143 | 0.564977572 | 2.34E-07 |
| International flight arrivals | Expenditure | 6.080921908 | 0.543025275 | 8.30E-07 |
| International flight arrivals | HIV | 3.482680036 | 0.406234728 | 0.000329094 |
| MPXV biodiversity | Education | 3.429384684 | -0.400366613 | 0.000372062 |
| MPXV biodiversity | Expenditure | 6.391473966 | -0.542836748 | 4.06E-07 |
| MSM population size | COVID-19 | 5.958607315 | 0.637537994 | 1.10E-06 |
| MSM population size | Air passengers | 5.187755303 | 0.622291131 | 6.49E-06 |
| MSM population size | Inbound travelers | 2.244248288 | 0.457703081 | 0.005698384 |
| Precipitation | Overweight | 3.1787584 | -0.389351316 | 0.000662585 |
| Precipitation | Low physical activity | 6.180456064 | -0.537524893 | 6.60E-07 |
| Same sex marriage | Expenditure | 5.677780705 | 0.513532595 | 2.10E-06 |
| Same sex marriage | HAQ | 3.720353585 | 0.415397086 | 0.000190391 |
| Same sex marriage | MSM population size | 2.392806705 | 0.407458829 | 0.00404756 |
| Same sex marriage | GDP | 3.29633211 | 0.387098903 | 0.000505438 |
| Same sex marriage | International flight arrivals | 2.607370042 | 0.342342163 | 0.002469619 |
| Same sex marriage | IHR | 2.351596854 | 0.322833119 | 0.004450442 |
| Same sex marriage | Air passengers | 2.106078416 | 0.31759678 | 0.007832882 |
| Same sex marriage | MPXV biodiversity | 4.161150909 | -0.429882732 | 6.90E-05 |
| Temperature | Expenditure | 3.595881577 | -0.415821423 | 0.000253582 |
| Temperature | HAQ | 5.982966661 | -0.535764702 | 1.04E-06 |
| Temperature | Education | 6.826813732 | -0.572458047 | 1.49E-07 |

MPX, monkeypox; GDP, gross domestic product; MSM, men who have sex with men; MPXV, monkeypox virus; HIV, Human Immunodeficiency Virus; COVID-19, coronavirus disease 2019; HAQ, Healthcare Access and Quality; IHR, International Health Regulations

| B: Variable in all infected countries in 2022 | | Weight | Correlation r | P value |
| --- | --- | --- | --- | --- |
| Monkeypox | International flight arrivals | 10.2362924 | 0.639583272 | 5.80E-11 |
| Monkeypox | COVID-19 | 8.03133671 | 0.56841911 | 9.30E-09 |
| Monkeypox | Expenditure | 5.59521172 | 0.487663744 | 2.54E-06 |
| Monkeypox | Same sex marriage | 5.2853286 | 0.464354547 | 5.18E-06 |
| Monkeypox | Inbound travelers | 4.87691102 | 0.53398065 | 1.33E-05 |
| Monkeypox | MSM population size | 4.32749567 | 0.515941634 | 4.70E-05 |
| Monkeypox | GDP | 3.53162615 | 0.383266663 | 0.00029402 |
| Monkeypox | HAQ | 3.47682188 | 0.384390356 | 0.00033356 |
| Monkeypox | Air passengers | 3.03455231 | 0.377200899 | 0.00092352 |
| Monkeypox | Education | 2.30243529 | 0.305455565 | 0.00498385 |
| Monkeypox | Aged under 40 | 2.24286768 | -0.30264396 | 0.00571653 |
| Aged under 40 | HAQ | 13.7241409 | -0.73162805 | 1.89E-14 |
| Aged under 40 | Education | 11.1329453 | -0.67386911 | 7.36E-12 |
| Aged under 40 | Expenditure | 10.3280031 | -0.65417253 | 4.70E-11 |
| Aged under 40 | Temperature | 7.53752153 | 0.575438169 | 2.90E-08 |
| Aged under 40 | COVID-19 | 7.13727708 | -0.55560072 | 7.29E-08 |
| Aged under 40 | International flight arrivals | 5.44362465 | -0.49183364 | 3.60E-06 |
| Aged under 40 | MPXV biodiversity | 4.77779998 | 0.456000105 | 1.67E-05 |
| Aged under 40 | IHR | 3.52067428 | -0.39379712 | 0.00030153 |
| Aged under 40 | HIV | 3.2459489 | 0.374728997 | 0.00056761 |
| Aged under 40 | Inbound travelers | 2.38910825 | -0.38124098 | 0.00408218 |
| Air passengers | COVID-19 | 11.0263476 | 0.691097848 | 9.41E-12 |
| Air passengers | Inbound travelers | 5.9923252 | 0.623585847 | 1.02E-06 |
| Education | Expenditure | 14 | 0.791158642 | 0 |
| Education | COVID-19 | 5.22383975 | 0.474088402 | 5.97E-06 |
| Education | Overweight | 2.28893628 | 0.304417445 | 0.00514119 |
| Expenditure | COVID-19 | 6.06093131 | 0.506806251 | 8.69E-07 |
| Expenditure | Air passengers | 3.05364152 | 0.378493254 | 0.00088381 |
| Forest area | Precipitation | 5.31327127 | 0.478065572 | 4.86E-06 |
| Forest area | MPXV biodiversity | 2.51575341 | 0.31587504 | 0.00304963 |
| GDP | HAQ | 14 | 0.921352134 | 0 |
| GDP | Education | 14 | 0.793685732 | 0 |
| GDP | Expenditure | 14 | 0.965791559 | 0 |
| GDP | IHR | 11.4424927 | 0.671755382 | 3.61E-12 |
| GDP | MPXV biodiversity | 10.9873414 | -0.65512498 | 1.03E-11 |
| GDP | Aged under 40 | 9.16868989 | -0.6199187 | 6.78E-10 |
| GDP | International flight arrivals | 8.47851469 | 0.59660102 | 3.32E-09 |
| GDP | Temperature | 4.12931699 | -0.42329753 | 7.42E-05 |
| GDP | HIV | 4.11060293 | -0.41757619 | 7.75E-05 |
| GDP | COVID-19 | 4.00870411 | 0.412149438 | 9.80E-05 |
| GDP | Overweight | 3.62228774 | 0.392904751 | 0.00023862 |
| GDP | Air passengers | 3.0134818 | 0.375768257 | 0.00096943 |
| HAQ | Education | 14 | 0.765096606 | 0 |
| HAQ | Expenditure | 14 | 0.935149114 | 0 |
| HAQ | MPXV biodiversity | 9.25465358 | -0.61629601 | 5.56E-10 |
| HAQ | International flight arrivals | 6.99514424 | 0.55346981 | 1.01E-07 |
| HAQ | IHR | 6.74269537 | 0.541330622 | 1.81E-07 |
| HAQ | COVID-19 | 4.49303834 | 0.4395827 | 3.21E-05 |
| HAQ | HIV | 4.4190967 | -0.4408196 | 3.81E-05 |
| HAQ | Overweight | 3.38999519 | 0.383625923 | 0.00040739 |
| HAQ | Air passengers | 2.52747817 | 0.343032136 | 0.0029684 |
| HIV | MSM population size | 6.03081124 | 0.606277056 | 9.32E-07 |
| HIV | Inbound travelers | 4.24032546 | 0.506805808 | 5.75E-05 |
| HIV | Education | 3.5958388 | -0.39362683 | 0.00025361 |
| HIV | Air passengers | 3.1744391 | 0.389065441 | 0.00066921 |
| HIV | Expenditure | 3.14719867 | -0.36419737 | 0.00071253 |
| HIV | COVID-19 | 2.45117763 | 0.312995896 | 0.00353853 |
| IHR | Expenditure | 9.90642464 | 0.631034183 | 1.24E-10 |
| IHR | Air passengers | 9.41996014 | 0.64963668 | 3.80E-10 |
| IHR | COVID-19 | 8.91437127 | 0.603633171 | 1.22E-09 |
| IHR | Education | 5.70931842 | 0.49510332 | 1.95E-06 |
| IHR | MSM population size | 3.33362711 | 0.452430254 | 0.00046385 |
| IHR | Inbound travelers | 1.99798872 | 0.34129667 | 0.01004642 |
| Inbound travelers | COVID-19 | 7.6705149 | 0.656956535 | 2.14E-08 |
| International flight arrivals | COVID-19 | 14 | 0.805333557 | 0 |
| International flight arrivals | Air passengers | 13.9908019 | 0.759822168 | 1.02E-14 |
| International flight arrivals | IHR | 10.7445122 | 0.664548267 | 1.80E-11 |
| International flight arrivals | Expenditure | 10.2065895 | 0.651070388 | 6.21E-11 |
| International flight arrivals | Inbound travelers | 9.75576999 | 0.734415584 | 1.75E-10 |
| International flight arrivals | MSM population size | 5.86620117 | 0.603518268 | 1.36E-06 |
| International flight arrivals | Education | 3.82879036 | 0.411674723 | 0.00014832 |
| International flight arrivals | Overweight | 3.45670138 | 0.389955576 | 0.00034938 |
| Low physical activity | COVID-19 | 2.42455751 | -0.31104163 | 0.00376221 |
| Low physical activity | MSM population size | 1.6603452 | -0.30865801 | 0.02186023 |
| MPXV biodiversity | Expenditure | 11.2264958 | -0.66374111 | 5.94E-12 |
| MPXV biodiversity | Education | 7.39902851 | -0.55890296 | 3.99E-08 |
| MPXV biodiversity | IHR | 4.81993368 | -0.45298858 | 1.51E-05 |
| MPXV biodiversity | Overweight | 3.70313743 | -0.39529624 | 0.00019809 |
| MPXV biodiversity | International flight arrivals | 3.14509239 | -0.36197814 | 0.00071599 |
| MPXV biodiversity | HIV | 2.36768093 | 0.305068735 | 0.00428864 |
| MPXV biodiversity | Air passengers | 2.21153424 | -0.31570173 | 0.00614421 |
| MSM population size | COVID-19 | 6.48984138 | 0.62125769 | 3.24E-07 |
| MSM population size | Air passengers | 5.80626273 | 0.625271053 | 1.56E-06 |
| MSM population size | Inbound travelers | 2.24424831 | 0.457703081 | 0.00569838 |
| Overweight | Expenditure | 4.00392198 | 0.411892468 | 9.91E-05 |
| Precipitation | Low physical activity | 4.99018384 | -0.46345661 | 1.02E-05 |
| Precipitation | Overweight | 3.85699286 | -0.41085928 | 0.000139 |
| Precipitation | MPXV biodiversity | 2.53983008 | 0.319413245 | 0.00288516 |
| Same sex marriage | Expenditure | 7.13089842 | 0.54680262 | 7.40E-08 |
| Same sex marriage | MPXV biodiversity | 5.58868278 | -0.47729763 | 2.58E-06 |
| Same sex marriage | HAQ | 5.00758376 | 0.464260684 | 9.83E-06 |
| Same sex marriage | GDP | 4.64027317 | 0.441967341 | 2.29E-05 |
| Same sex marriage | International flight arrivals | 3.68510706 | 0.394273182 | 0.00020649 |
| Same sex marriage | IHR | 3.55998605 | 0.387080822 | 0.00027543 |
| Same sex marriage | MSM population size | 2.97178417 | 0.425850397 | 0.00106713 |
| Same sex marriage | COVID-19 | 2.87926053 | 0.338957049 | 0.0013205 |
| Same sex marriage | Air passengers | 2.81688258 | 0.362078066 | 0.00152447 |
| Same sex marriage | Overweight | 2.34314246 | 0.306758185 | 0.00453793 |
| Temperature | Education | 9.47296056 | -0.63153331 | 3.37E-10 |
| Temperature | HAQ | 8.47839345 | -0.6026572 | 3.32E-09 |
| Temperature | Expenditure | 5.98788641 | -0.51205122 | 1.03E-06 |
| Temperature | MPXV biodiversity | 3.50136565 | 0.38150111 | 0.00031524 |
| Temperature | HIV | 2.88292014 | 0.347004744 | 0.00130942 |
| Temperature | COVID-19 | 2.3726598 | -0.30898046 | 0.00423975 |

MPX, monkeypox; GDP, gross domestic product; MSM, men who have sex with men; MPXV, monkeypox virus; HIV, Human Immunodeficiency Virus; COVID-19, coronavirus disease 2019; HAQ, Healthcare Access and Quality; IHR, International Health Regulations

| C: Variable in all infected countries during 2001-2021 | | Weight | Correlation r | P value |
| --- | --- | --- | --- | --- |
| Monkeypox | HIV | 2.458114 | 0.794529 | 0.003482 |
| Monkeypox | Population density | 1.509597 | -0.648409 | 0.030932 |
| Aged under 40 | Air passenger | 1.43708 | -0.738095 | 0.036553 |
| Aged under 40 | MPXV Biodiversity | 1.897685 | 0.718963 | 0.012657 |
| Aged under 40 | COVID-19 | 1.452385 | -0.636364 | 0.035287 |
| Aged under 40 | Expenditure | 2.681281 | -0.818182 | 0.002083 |
| Aged under 40 | HAQ | 3.481264 | -0.881818 | 0.00033 |
| Aged under 40 | IHR | 2.426423 | -0.790909 | 0.003746 |
| Aged under 40 | Overweight | 2.786004 | -0.854545 | 0.001637 |
| Aged under 40 | Education | 1.731304 | -0.690909 | 0.018565 |
| Air passenger | Expenditure | 3.064005 | 0.928571 | 0.000863 |
| Air passenger | Overweight | 1.44083 | 0.785714 | 0.036238 |
| Air passenger | Education | 1.332283 | 0.714286 | 0.046528 |
| MPXV Biodiversity | Air passenger | 2.746738 | -0.908403 | 0.001792 |
| MPXV Biodiversity | Expenditure | 2.800141 | -0.829572 | 0.001584 |
| MPXV Biodiversity | Overweight | 1.415628 | -0.658539 | 0.038404 |
| MPXV Biodiversity | Education | 1.347366 | -0.612962 | 0.04494 |
| COVID-19 | Air passenger | 3.584359 | 0.952381 | 0.00026 |
| COVID-19 | MPXV Biodiversity | 2.703331 | -0.820355 | 0.00198 |
| COVID-19 | Expenditure | 2.50718 | 0.8 | 0.00311 |
| COVID-19 | Overweight | 1.600384 | 0.69697 | 0.025097 |
| COVID-19 | Education | 2.980808 | 0.845455 | 0.001045 |
| Forest area | MPXV Biodiversity | 2.21606 | 0.76505 | 0.006081 |
| Forest area | COVID-19 | 1.539634 | -0.654545 | 0.028865 |
| Forest area | Expenditure | 1.892804 | -0.718182 | 0.0128 |
| Forest area | HAQ | 1.783281 | -0.7 | 0.016471 |
| GDP | Aged under 40 | 3.976363 | -0.909091 | 0.000106 |
| GDP | Air passenger | 1.992443 | 0.833333 | 0.010176 |
| GDP | MPXV Biodiversity | 2.402182 | -0.788094 | 0.003961 |
| GDP | COVID-19 | 2.205299 | 0.763636 | 0.006233 |
| GDP | Expenditure | 3.631791 | 0.890909 | 0.000233 |
| GDP | GDP growth | 1.330723 | -0.609091 | 0.046696 |
| GDP | HAQ | 4.400797 | 0.927273 | 0.00004 |
| GDP | IHR | 1.892804 | 0.718182 | 0.0128 |
| GDP | Overweight | 3.689365 | 0.915152 | 0.000204 |
| GDP | Education | 2.349385 | 0.781818 | 0.004473 |
| GDP growth | Aged under 40 | 1.632401 | 0.672727 | 0.023313 |
| GDP growth | HAQ | 1.370126 | -0.618182 | 0.042646 |
| GDP growth | Overweight | 1.664223 | -0.709091 | 0.021666 |
| HAQ | Air passenger | 3.064005 | 0.928571 | 0.000863 |
| HAQ | MPXV Biodiversity | 3.252262 | -0.866442 | 0.000559 |
| HAQ | COVID-19 | 2.50718 | 0.8 | 0.00311 |
| HAQ | Expenditure | 6.288853 | 0.972727 | 0.000001 |
| HAQ | Overweight | 2.786004 | 0.854545 | 0.001637 |
| HAQ | Education | 1.539634 | 0.654545 | 0.028865 |
| IHR | Air passenger | 2.185086 | 0.857143 | 0.00653 |
| IHR | MPXV Biodiversity | 1.897685 | -0.718963 | 0.012657 |
| IHR | COVID-19 | 2.275766 | 0.772727 | 0.005299 |
| IHR | Expenditure | 2.137747 | 0.754545 | 0.007282 |
| IHR | HAQ | 2.205299 | 0.763636 | 0.006233 |
| IHR | Overweight | 1.600384 | 0.69697 | 0.025097 |
| IHR | Education | 1.585292 | 0.663636 | 0.025984 |
| MSM | COVID-19 | 1.609875 | 0.733333 | 0.024554 |
| MSM | HIV | 1.302185 | 0.666667 | 0.049867 |
| MSM | IHR | 1.372201 | 0.683333 | 0.042442 |
| Overweight | Expenditure | 3.089449 | 0.878788 | 0.000814 |
| Population density | IHR | 1.410662 | 0.627273 | 0.038845 |
| Population density | Low physical activity | 1.452385 | -0.636364 | 0.035287 |
| Same sex marriage | Air passenger | 1.522593 | 0.755929 | 0.03002 |
| Same sex marriage | MPXV Biodiversity | 1.311602 | -0.604586 | 0.048798 |
| Same sex marriage | COVID-19 | 1.622395 | 0.67082 | 0.023856 |
| Same sex marriage | Expenditure | 1.622395 | 0.67082 | 0.023856 |
| Same sex marriage | HAQ | 1.622395 | 0.67082 | 0.023856 |
| Education | Expenditure | 1.452385 | 0.636364 | 0.035287 |
| Education | Overweight | 2.418515 | 0.818182 | 0.003815 |

MPX, monkeypox; GDP, gross domestic product; MSM, men who have sex with men; MPXV, monkeypox virus; HIV, Human Immunodeficiency Virus; COVID-19, coronavirus disease 2019; HAQ, Healthcare Access and Quality; IHR, International Health Regulations

# Supplementary Table 6. Parameters used in the SEIR model

| Variable/ parameter | description | value | type |
| --- | --- | --- | --- |
| $S_{i}$ | Susceptible individuals at continent i |  | Time series |
| $E_{i}$ | Exposed individuals at continent i |  | Time series |
| $I_{i}$ | Infected individuals at continent i |  | Time series |
| $R_{i}$ | Recovered individuals at continent i |  | Time series |
| $S_{\mathrm{ij}}$ | Susceptible individuals flying from continent i to continent j |  | Time series |
| $E_{\mathrm{ij}}$ | Exposed individuals flying from continent i to continent j |  | Time series |
| $I_{\mathrm{ij}}$ | Infected individuals flying from continent i to continent j |  | Time series |
| $R_{\mathrm{ij}}$ | Recovered individuals flying from continent i to continent j |  | Time series |
| $\mathrm{Passengers}_{\mathrm{ij}}$ | Passengers flying from continent i to continent j |  | Time series |
| $N_{i}$ | Total population at continent i |  | constant |
| $n_{i}$ | Adjusted starting value of Susceptible population at continent i | Population under 40 at continent i + (1/5.2)*population above 40 at continent i | constant |
| $\beta_{i1}$ | Transmission rate, i.e. number of transmissions per time: $S_{i}$to $E_{i}$ from January 31 to June 23 |  | fitted |
| $\beta_{i2}$ | Transmission rate, i.e. number of transmissions per time: $S_{i}$to $E_{i}$ from June 23 |  | fitted |
| $\sigma$ | Incubation rate: $E_{i}$ to $I_{i}$ | 1/13 | constant |
| $\gamma$ | Recovery rate:$I_{i}$to $R_{i}$ | 1/21 | constant |
| ${R0}_{i1}/{R0}_{i2}$ | The population reproduction number at continent i | $\frac{\beta_{i1}}{\gamma}/\frac{\beta_{i2}}{\gamma}$ | fitted |

# Supplementary Table 7. Assessment of MPX risks on the country level

| Country | Country risk | MPX case | Continents | R0 (C) | R0 (E) |
| --- | --- | --- | --- | --- | --- |
| United States | High | 11843 | Northern America | 3.51 | 7.841 |
| Spain | High | 5719 | Europe | 1.349 | 7.017 |
| Germany | High | 3142 | Europe | 1.349 | 7.017 |
| United Kingdom | High | 3023 | Europe | 1.349 | 7.017 |
| France | High | 2663 | Europe | 1.349 | 7.017 |
| Canada | High | 1058 | Northern America | 3.51 | 7.841 |
| Netherlands | High | 1025 | Europe | 1.349 | 7.017 |
| Portugal | High | 770 | Europe | 1.349 | 7.017 |
| Italy | High | 644 | Europe | 1.349 | 7.017 |
| Belgium | High | 546 | Europe | 1.349 | 7.017 |
| Switzerland | High | 387 | Europe | 1.349 | 7.017 |
| Austria | High | 198 | Europe | 1.349 | 7.017 |
| Denmark | High | 151 | Europe | 1.349 | 7.017 |
| Sweden | High | 126 | Europe | 1.349 | 7.017 |
| Ireland | High | 101 | Europe | 1.349 | 7.017 |
| Norway | High | 74 | Europe | 1.349 | 7.017 |
| Australia | High | 71 | Oceania | 1.666 | 7.243 |
| Luxembourg | High | 41 | Europe | 1.349 | 7.017 |
| Finland | High | 22 | Europe | 1.349 | 7.017 |
| Singapore | High | 15 | Asia | 0.695 | 3.679 |
| Iceland | High | 11 | Europe | 1.349 | 7.017 |
| New Zealand | High | 4 | Oceania | 1.666 | 7.243 |
| Japan | High | 4 | Asia | 0.695 | 3.679 |
| Korea, Rep. | High | 1 | Asia | 0.695 | 3.679 |
| Bolivia | Low | 15 | Latin America | 4.132 | 7.69 |
| India | Low | 10 | Asia | 0.695 | 3.679 |
| South Africa | Low | 4 | Africa | 1.378 | 1.605 |
| Guatemala | Low | 3 | Latin America | 4.132 | 7.69 |
| Honduras | Low | 3 | Latin America | 4.132 | 7.69 |
| Sudan | Low | 1 | Africa | 1.378 | 1.605 |
| Haiti | Low | 0 | Latin America | 4.132 | 7.69 |
| Kiribati | Low | 0 | Oceania | 1.666 | 7.243 |
| Papua New Guinea | Low | 0 | Oceania | 1.666 | 7.243 |
| Solomon Islands | Low | 0 | Oceania | 1.666 | 7.243 |
| Vanuatu | Low | 0 | Oceania | 1.666 | 7.243 |
| Afghanistan | Low | 0 | Asia | 0.695 | 3.679 |
| Bangladesh | Low | 0 | Asia | 0.695 | 3.679 |
| Bhutan | Low | 0 | Asia | 0.695 | 3.679 |
| Iraq | Low | 0 | Asia | 0.695 | 3.679 |
| Cambodia | Low | 0 | Asia | 0.695 | 3.679 |
| Lao PDR | Low | 0 | Asia | 0.695 | 3.679 |
| Maldives | Low | 0 | Asia | 0.695 | 3.679 |
| Myanmar | Low | 0 | Asia | 0.695 | 3.679 |
| Nepal | Low | 0 | Asia | 0.695 | 3.679 |
| Pakistan | Low | 0 | Asia | 0.695 | 3.679 |
| Angola | Low | 0 | Africa | 1.378 | 1.605 |
| Burundi | Low | 0 | Africa | 1.378 | 1.605 |
| Burki Faso | Low | 0 | Africa | 1.378 | 1.605 |
| Comoros | Low | 0 | Africa | 1.378 | 1.605 |
| CaboVerde | Low | 0 | Africa | 1.378 | 1.605 |
| Djibouti | Low | 0 | Africa | 1.378 | 1.605 |
| Ethiopia | Low | 0 | Africa | 1.378 | 1.605 |
| Guinea | Low | 0 | Africa | 1.378 | 1.605 |
| Gambia, The | Low | 0 | Africa | 1.378 | 1.605 |
| Equatorial Guinea | Low | 0 | Africa | 1.378 | 1.605 |
| Kenya | Low | 0 | Africa | 1.378 | 1.605 |
| Lesotho | Low | 0 | Africa | 1.378 | 1.605 |
| Madagascar | Low | 0 | Africa | 1.378 | 1.605 |
| Mali | Low | 0 | Africa | 1.378 | 1.605 |
| Mozambique | Low | 0 | Africa | 1.378 | 1.605 |
| Mauritania | Low | 0 | Africa | 1.378 | 1.605 |
| Malawi | Low | 0 | Africa | 1.378 | 1.605 |
| Niger | Low | 0 | Africa | 1.378 | 1.605 |
| Rwanda | Low | 0 | Africa | 1.378 | 1.605 |
| Senegal | Low | 0 | Africa | 1.378 | 1.605 |
| Eswatini | Low | 0 | Africa | 1.378 | 1.605 |
| Chad | Low | 0 | Africa | 1.378 | 1.605 |
| Togo | Low | 0 | Africa | 1.378 | 1.605 |
| Uganda | Low | 0 | Africa | 1.378 | 1.605 |
| Zambia | Low | 0 | Africa | 1.378 | 1.605 |
| Zimbabwe | Low | 0 | Africa | 1.378 | 1.605 |
| Brazil | Medium | 2893 | Latin America | 4.132 | 7.69 |
| Peru | Medium | 775 | Latin America | 4.132 | 7.69 |
| Israel | Medium | 189 | Asia | 0.695 | 3.679 |
| Mexico | Medium | 147 | Latin America | 4.132 | 7.69 |
| Chile | Medium | 141 | Latin America | 4.132 | 7.69 |
| Colombia | Medium | 129 | Latin America | 4.132 | 7.69 |
| Poland | Medium | 85 | Europe | 1.349 | 7.017 |
| Hungary | Medium | 51 | Europe | 1.349 | 7.017 |
| Argenti | Medium | 49 | Latin America | 4.132 | 7.69 |
| Greece | Medium | 48 | Europe | 1.349 | 7.017 |
| Czech Republic | Medium | 35 | Europe | 1.349 | 7.017 |
| Romania | Medium | 31 | Europe | 1.349 | 7.017 |
| Malta | Medium | 30 | Europe | 1.349 | 7.017 |
| Serbia | Medium | 23 | Europe | 1.349 | 7.017 |
| Ecuador | Medium | 16 | Latin America | 4.132 | 7.69 |
| Croatia | Medium | 12 | Europe | 1.349 | 7.017 |
| Estonia | Medium | 9 | Europe | 1.349 | 7.017 |
| Lebanon | Medium | 6 | Asia | 0.695 | 3.679 |
| Lithuania | Medium | 5 | Europe | 1.349 | 7.017 |
| Saudi Arabia | Medium | 5 | Asia | 0.695 | 3.679 |
| Turkey | Medium | 5 | Asia | 0.695 | 3.679 |
| Jamaica | Medium | 4 | Latin America | 4.132 | 7.69 |
| Bulgaria | Medium | 4 | Europe | 1.349 | 7.017 |
| Thailand | Medium | 4 | Asia | 0.695 | 3.679 |
| Costa Rica | Medium | 3 | Latin America | 4.132 | 7.69 |
| Latvia | Medium | 3 | Europe | 1.349 | 7.017 |
| China | Medium | 3 | Asia | 0.695 | 3.679 |
| Cyprus | Medium | 3 | Asia | 0.695 | 3.679 |
| Qatar | Medium | 3 | Asia | 0.695 | 3.679 |
| Pama | Medium | 2 | Latin America | 4.132 | 7.69 |
| Uruguay | Medium | 2 | Latin America | 4.132 | 7.69 |
| Barbados | Medium | 1 | Latin America | 4.132 | 7.69 |
| Venezuela, RB | Medium | 1 | Latin America | 4.132 | 7.69 |
| Bosnia and Herzegovi | Medium | 1 | Europe | 1.349 | 7.017 |
| Montenegro | Medium | 1 | Europe | 1.349 | 7.017 |
| Russian Federation | Medium | 1 | Europe | 1.349 | 7.017 |
| Georgia | Medium | 1 | Asia | 0.695 | 3.679 |
| Philippines | Medium | 1 | Asia | 0.695 | 3.679 |
| Morocco | Medium | 1 | Africa | 1.378 | 1.605 |
| Bahamas, The | Medium | 0 | Latin America | 4.132 | 7.69 |
| Belize | Medium | 0 | Latin America | 4.132 | 7.69 |
| Cuba | Medium | 0 | Latin America | 4.132 | 7.69 |
| Dominica | Medium | 0 | Latin America | 4.132 | 7.69 |
| Guya | Medium | 0 | Latin America | 4.132 | 7.69 |
| Nicaragua | Medium | 0 | Latin America | 4.132 | 7.69 |
| Paraguay | Medium | 0 | Latin America | 4.132 | 7.69 |
| El Salvador | Medium | 0 | Latin America | 4.132 | 7.69 |
| Surime | Medium | 0 | Latin America | 4.132 | 7.69 |
| Trinidad and Tobago | Medium | 0 | Latin America | 4.132 | 7.69 |
| Micronesia, Fed. Sts. | Medium | 0 | Oceania | 1.666 | 7.243 |
| Tonga | Medium | 0 | Oceania | 1.666 | 7.243 |
| Ukraine | Medium | 0 | Europe | 1.349 | 7.017 |
| Armenia | Medium | 0 | Asia | 0.695 | 3.679 |
| Azerbaijan | Medium | 0 | Asia | 0.695 | 3.679 |
| Bahrain | Medium | 0 | Asia | 0.695 | 3.679 |
| Indonesia | Medium | 0 | Asia | 0.695 | 3.679 |
| Iran, Islamic Rep. | Medium | 0 | Asia | 0.695 | 3.679 |
| Kazakhstan | Medium | 0 | Asia | 0.695 | 3.679 |
| Kuwait | Medium | 0 | Asia | 0.695 | 3.679 |
| Sri Lanka | Medium | 0 | Asia | 0.695 | 3.679 |
| Mongolia | Medium | 0 | Asia | 0.695 | 3.679 |
| Malaysia | Medium | 0 | Asia | 0.695 | 3.679 |
| Oman | Medium | 0 | Asia | 0.695 | 3.679 |
| Tajikistan | Medium | 0 | Asia | 0.695 | 3.679 |
| Uzbekistan | Medium | 0 | Asia | 0.695 | 3.679 |
| Vietnam | Medium | 0 | Asia | 0.695 | 3.679 |
| Egypt, Arab Rep. | Medium | 0 | Africa | 1.378 | 1.605 |
| Mauritius | Medium | 0 | Africa | 1.378 | 1.605 |
| Seychelles | Medium | 0 | Africa | 1.378 | 1.605 |
| Tunisia | Medium | 0 | Africa | 1.378 | 1.605 |

E, Early stage is the period before 23 June, 2022; C: the current stage is the period after 23 June, 2022. R0: basic reproduction number

**Supplementary File 1. MPXV biodiversity**

MPXV biodiversity was evaluated by the list of 59 species was then compared against known species ranges, using the International Union for Conservation of Nature (IUCN) Red List of Threatened Species (reference, lancet covid-19). After that, we counted via ArcMap 10.8, at each of the grid cells of a 0.25 decimal degree grid mesh that cover the global land, the number of MPXV infected species whose distribution ranges covered that grid cell. Then, the number of infected species per grid cell was summarized over each country, leading to the mean and median species richness of mammal species that can be infected by the MPVX global (Sfigure 1 and Stable 3)

**Supplementary File 2. Grey model**

The original Grey model with a 1-variable and 1-order equation GM (1,1)

Assuming that the original sequence shown as:

$$X^{(0)}=\left\{ X^{\left( 0 \right)}\left( i \right), i=1,2,3,\ldots,n \right\}$$

Performing an accumulation to generate a new sequence:

$$X^{(1)}=\left\{ X^{\left( 1 \right)}\left( k \right),k=1,2,3,\ldots, n \right\}$$

Solving adjacent neighbor means by the following formula:

$$Z^{\left( 1 \right)}\left( t \right)=\frac{1}{2}\left[ X^{\left( 1 \right)}\left( t \right)+X^{\left( 1 \right)}\left( t+1 \right) \right], t=1, 2, 3\ldots, n$$

Establishing first-order linear differential equations by the following formula:

$$\frac{{dX}^{\left( 1 \right)}(t)}{dt}+{ax}^{\left( 1 \right)}\left( t \right)=u$$

Assuming that $\hat{a}={(a, b)}^{T}$ then identification a can be calculated by the following formula:

$$\hat{a}={(B^{T}B)}^{-1}B^{T}Y$$

$$Y=\left[ X^{\left( 0 \right)}\left( 2 \right),X^{\left( 0 \right)}\left( 3 \right),\ldots X^{\left( 0 \right)}(n) \right]^{T}$$

$$B=\left[ \begin{matrix} {-Z}^{\left( 1 \right)}(2) & 1 \\ {-Z}^{\left( 1 \right)}\left( 3 \right) & 1 \\ \begin{matrix} \vdots\\ -Z^{\left( 1 \right)}(n) \end{matrix} & \begin{matrix} \vdots\\ 1 \end{matrix} \end{matrix} \right]$$

The GM(1,1) model can be expressed as:

$$\hat{X}^{(1)}\left( g+1 \right)=\frac{b}{a}+\left[ X^{\left( 0 \right)}\left( 1 \right)-\frac{b}{a} \right]e^{-ag} g=1,2,3\ldots n$$

where a is the development coefficient, b is the grey effect. Model test evaluated by the posterior error test.


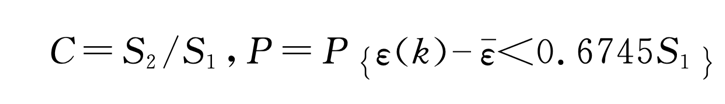


The coefficients of GM (1, 1)

| coefficient | a | b | C | p |
| --- | --- | --- | --- | --- |
| Value | -0.0066 | 31673.0012 | 0.2634 | 0.800 |

The criteria of GM (1,1) prediction length

| Development coefficient a | Prediction length |
| --- | --- |
| -a ≤ 0.3 | Medium-and long-term prediction |
| 0.3 < -a < 0.5 | Short-term prediction |
| 0.5 < -a < 1.0 | Modified model to predict |
| 1.0< -a | Not suitable for grey prediction model |

Criteria for GM ( 1,1 ) prediction accuracy

| Prediction accuracy grade | Mean square deviation ratio C | Small probability of error P |
| --- | --- | --- |
| Excellent | ≤0.35 | ≥0.95 |
| Qualified | ≤0.50 | ≥0.80 |
| Barely qualified | ≤0.65 | ≥0.70 |
| Unqualified | >0.65 | <0.70 |

**Supplementary File 3. SEIR model**

**1) Standard SEIR**

SEIR is a standard compartmental model commonly used for simulation and prediction of epidemics. Population is divided into different groups based on infection status: S—Susceptible population, E—Exposed (infected but not yet infectious) population, I—Infectious population, R—Recovered population, with $\beta$, $\sigma$, $\gamma$ representing transmission, incubation, recovery rate. Our standard SEIR is as follows.

$$\frac{dS}{dt}=-\frac{\beta SI}{N}$$

$$\frac{dE}{dt}=\frac{\beta SI}{N}-\sigma E$$

$$\frac{dI}{dt}=\sigma E-\gamma I$$

$$\frac{dR}{dt}=\gamma I$$

**2)** **Improved SEIR**

**a. Including international mobility**

Human mobility is likely to be an important cause of disease transmission in the early stages of MPX outbreak in 2022. We took the mobility of pre-symptomatic cases into consideration, and incorporated international flight arrivals data in the standard SEIR. We considered six continents (Europe, Asia, Africa, Latin America, North America and Oceania), and for each continental population, we assume that people are free to travel in and out. The number of international flight arrivals by continent from 31 January 2022 to 31 December 2022 was simulated by the number of international flight arrivals from 1 April to 31 May: data for other dates were simulated according to the weekly cycle; the departure and arrival continents were identified and the average number of passengers of every Monday within April 1 to May 31 were calculated, which would give the data for every other Monday, and similar for the other weekdays. Our improved SEIR is as follows.

$$\begin{aligned} &\frac{dS_{i}}{dt}=-\frac{\beta_{i}S_{i}I_{i}}{N_{i}}+\sum_{i} S_{ji}-\sum_{i} S_{ij} \\ &\frac{dE_{i}}{dt}=\frac{\beta_{i}S_{i}I_{i}}{N_{i}}-\sigma E_{i}+\sum_{i} E_{ji}-\sum_{i} E_{ij} \\ &\frac{dI_{i}}{dt}=\sigma E_{i}-\gamma I_{i}+\sum_{i} I_{ji}-\sum_{i} I_{ij} \\ &\frac{dR_{i}}{dt}=\gamma I_{i}+\sum_{i} R_{ji}-\sum_{i} R_{ij} \end{aligned}$$

Further assumptions are:

1. Individuals in the infected compartment do not travel across populations. Infected individuals develop severe symptoms such as rash, which seriously affect patients’ daily life, and are easily identifiable. Moreover, passengers with fever, which is one of the symptoms of Monkeypox, would very likely be denied boarding during the post-COVID.^1^ Therefore for each continent i, we set $I_{ij}$ and $I_{ji}$ to 0 (any j).
2. Since what we consider is the early stage of the current pandemic, the number of recovered individuals that travel across populations is negligible, which hardly affect the transmission of disease. Therefore, for each continent, we set $R_{ij}$ and $R_{ji}$ to 0 (any j).

With the assumptions above, and that people are supposed to freely travel across the populations, our SEIR equations can be rewritten as:

$$\begin{aligned} &\frac{dS_{i}}{dt}=-\frac{\beta_{i}S_{i}I_{i}}{N_{i}}+\sum_{i} S_{ji}-\sum_{i} S_{ij} \\ &\frac{dE_{i}}{dt}=\frac{\beta_{i}S_{i}I_{i}}{N_{i}}-\sigma E_{i}+\sum_{i} E_{ji}-\sum_{i} E_{ij} \\ &\frac{dI_{i}}{dt}=\sigma E_{i}-\gamma I_{i} \\ &\frac{dR_{i}}{dt}=\gamma I_{i} \end{aligned}$$

$$S_{ij}={Passengers}_{ij}\times\frac{S_{i}}{N_{i}}$$

$$E_{ij}={Passengers}_{ij}\times\frac{E_{i}}{N_{i}}$$

**b. Adjusting Parameters**

$\boldsymbol{starting values of}\boldsymbol{S}_{\boldsymbol{i}}\boldsymbol{,}\boldsymbol{E}_{\boldsymbol{i}}\boldsymbol{,}\boldsymbol{I}_{\boldsymbol{i}}\mathbf{,}\boldsymbol{R}_{\boldsymbol{i}}$

It was reported that for people vaccinated smallpox, the chance that they get Monkeypox would be 1/5.2 times of those without vaccination. And since smallpox vaccine was stopped by 1980s, we assume that population over 40 years old were vaccinated, while population under 40 years old were not^2^. Therefore, we adjusted the initial value (and maximum value as time changes) of susceptible individuals at continent i ($S_{i}$) to

$$n_{i}=Population under 40 at continent i + (1/5.2)*population above 40 at continent i$$

The simulation of our modified SEIR starts from January 31, when cases were only reported in Africa. By January 31, 3 cases in Africa were confirmed (so we set starting value of $I_{j}$to 3, where continent j is Africa). Considering the reproduction number R0 of Monkeypox was reported to be around 2 ^3^,we set $E_{j}$ to 2*3=6. Starting values of other continents were set 0, since no case was reported until May 6.

$$\boldsymbol{\sigma and \gamma}$$

Since WHO reported that the latent period of Monkeypox ranges from 5 to 21 days, while the recovery period is 2-4 weeks ^4^, we took the medium and set incubation rate $\sigma=1/13$ (reciprocal of latent period), and recovery rate $\gamma=1/21$ (reciprocal of recovery period).

$$\boldsymbol{\beta}_{\boldsymbol{i1}}\boldsymbol{and}\boldsymbol{\beta}_{\boldsymbol{i2}}$$

The IHR Emergency Committee on the multi-country outbreak of monkeypox held its first meeting on 23 June 2022, to consider classifying monkeypox as an emergency of international concern Thus, by this time, monkeypox had become a widespread global concern and we considered that its transmission rate changed. We fitted two-phase $\beta$’s taking June 23 as the node. For continent i, we denote its transmission rate before June 23 by $\beta_{i1}$, and that after June 23 by $\beta_{i2}$

In conclusion, our improved SEIR model is as follows:

If earlier than June 23:

$$\begin{aligned} &\frac{dS_{i}}{dt}=-\frac{\beta_{i1}S_{i}I_{i}}{n_{i}}+\sum_{i} S_{ji}-\sum_{i} S_{ij} \\ &\frac{dE_{i}}{dt}=\frac{\beta_{i1}S_{i}I_{i}}{n_{i}}-\sigma E_{i}+\sum_{i} E_{ji}-\sum_{i} E_{ij} \\ &\frac{dI_{i}}{dt}=\sigma E_{i}-\gamma I_{i} \\ &\frac{dR_{i}}{dt}=\gamma I_{i} \end{aligned}$$

Else:

$$\begin{aligned} &\frac{dS_{i}}{dt}=-\frac{\beta_{i2}S_{i}I_{i}}{n_{i}}+\sum_{i} S_{ji}-\sum_{i} S_{ij} \\ &\frac{dE_{i}}{dt}=\frac{\beta_{i2}S_{i}I_{i}}{n_{i}}-\sigma E_{i}+\sum_{i} E_{ji}-\sum_{i} E_{ij} \\ &\frac{dI_{i}}{dt}=\sigma E_{i}-\gamma I_{i} \\ &\frac{dR_{i}}{dt}=\gamma I_{i} \end{aligned}$$

$$S_{ij}={Passengers}_{ij}\times\frac{S_{i}}{N_{i}}$$

$$E_{ij}={Passengers}_{ij}\times\frac{E_{i}}{N_{i}}$$

We fitted $\beta_{i1}, \beta_{i2}$ and derived the epidemic curve by minimizing mean square error (MSE). The loss function we minimized is:

$$\begin{aligned} &f\left( \beta_{i1},\beta_{i2} \right) \\ &=\sum_{i=1}^{6} \sum_{t=\text{ days from Jan }31}^{\mathrm{Aug} 16} \left( \frac{I_{i}\left( t \right)+R_{i}\left( t \right)-\text{ cumulative confirmed cases in continent }i(t)}{\text{ cumulative confirmed cases in continent }i (\text{Aug }16)} \right)^{2} \end{aligned}$$

# Reference

1. Ding X, Huang S, Leung A, Rabbany R. Incorporating dynamic flight network in SEIR to model mobility between populations. *Appl Netw Sci* 2021; **6**(1): 42.

2. Rimoin A, Mulembakani P, Johnston S, et al. Major increase in human monkeypox incidence 30 years after smallpox vaccination campaigns cease in the Democratic Republic of Congo. 2010; **107**(37): 16262-7.

3. Burki T. What does it mean to declare monkeypox a PHEIC? *The Lancet Infectious Diseases* 2022; **22**(9): 1286-7.

4. Gideon: Monkeypox: Global Status. <https://ebooks.gideononline.com/pdf?title=Monkeypox%3A+Global+Status&year=2022> (accessed 22-May 2022).
